# Adverse Cardiovascular Complications following prescription of programmed cell death 1 (PD-1) and programmed cell death ligand 1 (PD-L1) inhibitors: a propensity-score matched Cohort Study with competing risk analysis

**DOI:** 10.1186/s40959-021-00128-5

**Published:** 2022-03-17

**Authors:** Jiandong Zhou, Sharen Lee, Ishan Lakhani, Lei Yang, Tong Liu, Yuhui Zhang, Yunlong Xia, Wing Tak Wong, Kelvin King Hei Bao, Ian Chi Kei Wong, Gary Tse, Qingpeng Zhang

**Affiliations:** 1 Nuffield Department of Medicine, University of Oxford, Oxford, UK; 2 Cardio-Oncology Research Unit, Cardiovascular Analytics Group, UK Collaboration, Hong Kong, China; 3 Tianjin Key Laboratory of Ionic-Molecular Function of Cardiovascular Disease, Department of Cardiology, Tianjin Institute of Cardiology, Second Hospital of Tianjin Medical University, 300211 Tianjin, China; 4 Heart Failure Center, State Key Laboratory of Cardiovascular Disease, Fuwai Hospital, National Center for Cardiovascular Diseases, Chinese Academy of Medical Sciences and Peking Union Medical College, Beijing, China; 5 Department of Cardiology, The First Affiliated Hospital of Dalian Medical University, Dalian, China; 6 School of Life Sciences, Chinese University of Hong Kong, Hong Kong, China; 7 Department of Clinical Oncology, Queen Elizabeth Hospital, Hong Kong, China; 8 Department of Pharmacology and Pharmacy, University of Hong Kong, Pokfulam, Hong Kong China; 9 Kent and Medway Medical School, Canterbury, UK; 10 School of Data Science, City University of Hong Kong, Hong Kong, China

## Abstract

**Background:**

Programmed death-1 (PD-1) and programmed death- ligand 1 (PD-L1) inhibitors, such as pembrolizumab, nivolumab and atezolizumab, are major classes of immune checkpoint inhibitors that are increasingly used for cancer treatment. However, their use is associated with adverse cardiovascular events. We examined the incidence of new-onset cardiac complications in patients receiving PD-1 or PD-L1 inhibitors.

**Methods:**

Patients receiving PD-1 or PD-L1 inhibitors since their launch up to 31st December 2019 at publicly funded hospitals of Hong Kong, China, without pre-existing cardiac complications were included. The primary outcome was a composite of incident heart failure, acute myocardial infarction, atrial fibrillation, or atrial flutter with the last follow-up date of 31st December 2020. Propensity score matching between PD-L1 inhibitor use and PD-1 inhibitor use with a 1:2 ratio for patient demographics, past comorbidities and non-PD-1/PD-L1 medications was performed with nearest neighbour search strategy (0.1 caliper). Univariable and multivariable Cox regression analysis models were conducted. Competing risks models and multiple propensity matching approaches were considered for sensitivity analysis.

**Results:**

A total of 1959 patients were included. Over a median follow-up of 247 days (interquartile range [IQR]: 72-506), 320 (incidence rate [IR]: 16.31%) patients met the primary outcome after PD-1/PD-L1 treatment: 244 (IR: 12.57%) with heart failure, 38 (IR: 1.93%) with acute myocardial infarction, 54 (IR: 2.75%) with atrial fibrillation, 6 (IR: 0.31%) with atrial flutter. Compared with PD-1 inhibitor treatment, PD-L1 inhibitor treatment was significantly associated with lower risks of the composite outcome both before (hazard ratio [HR]: 0.32, 95% CI: [0.18-0.59], P value=0.0002) and after matching (HR: 0.34, 95% CI: [0.18-0.65], P value=0.001), and lower all-cause mortality risks before matching (HR: 0.77, 95% CI: [0.64-0.93], P value=0.0078) and after matching (HR: 0.80, 95% CI: [0.65-1.00], P value=0.0463). Patients who developed cardiac complications had shorter average readmission intervals and a higher number of hospitalizations after treatment with PD-1/PD-L1 inhibitors in both the unmatched and matched cohorts (P value<0.0001). Multivariable Cox regression models, competing risk analysis with cause-specific and subdistribution hazard models, and multiple propensity approaches confirmed these observations.

**Conclusions:**

Compared with PD-1 treatment, PD-L1 treatment was significantly associated with lower risk of new onset cardiac complications and all-cause mortality both before and after propensity score matching.

**Supplementary Information:**

The online version contains supplementary material available at 10.1186/s40959-021-00128-5.

## Introduction

The programmed death-1 (PD-1)/programmed death-ligand 1 (PD-L1) pathway is one of the major immune checkpoints for mitigating the immune response to prevent autoimmunity. The T-cell mediated pathway for cancer detection requires coinhibitory signal, in addition to the binding of T cell receptor on T cells. PD-1 and PD-L1, one of the best characterized co-inhibitory signaling pathway, control the magnitude and duration of response against autoimmunity [1]. However, cancer cells often devise strategies to hijack these mechanisms to evade anti-tumor immunity. In this regard, inhibitors of PD-1 (e.g., pembrolizumab, nivolumab, cemiplimab) and PD-L1 (e.g., atezolizumab, avelumab, durvalumab) have shown clinical efficacies against different types of solid tumors, including melanoma, non-small cell lung cancer (NSCLC), urothelial carcinoma and bladder cancer. Pembrolizumab is also the first agent to receive a “pan-cancer” approval by the United States Food and Drug Administration (FDA) for the treatment of unresectable or metastatic solid tumors that have high microsatellite instability or mismatch repair deficiency.

Despite their treatment efficacy in clinical oncology, immune-related adverse events associated with the use of immune checkpoint inhibitors (ICIs) are now increasingly recognized [2–5]. Adverse events include atherosclerosis, colitis, hepatitis, adrenocorticotropic hormone insufficiency, hypothyroidism, type 1 diabetes mellitus, and acute kidney injury [6–8]. To this end, cardiovascular complications are estimated to constitute approximately 2% of ICI-related adverse drug reactions [9]. The commonest is myocarditis, but other cardiovascular abnormalities reported are left ventricular dysfunction, acute myocardial infarction (AMI), cardiac arrhythmias and heart failure [10]. These cardiovascular complications typically present with clinical heterogeneity, and in turn account for the high morbidity and mortality rates observed in such patient cohorts. Whilst cardiotoxicity is being documented with an increasing frequency, their cumulative incidence rates remain largely unexplored. In this territory-wide study, we examined the incidence of cardiovascular events of incident heart failure, acute myocardial infarction, atrial fibrillation, or atrial flutter in cancer patients receiving PD-1 or PD-L1 inhibitors.

## Methods

### Study Population

This study was approved by The Joint Chinese University of Hong Kong - New Territories East Cluster Clinical Research Ethics Committee. Patients receiving PD-1 or PD-L1 inhibitors since their launch up to 31st December 2020 at publicly funded hospitals or their associated outpatient/ambulatory care facilities, without pre-existing cardiac complications (including heart failure, myocardial infarction, atrial fibrillation, and atrial flutter) were included. Patient data were obtained using the electronic health record database, which is connected to the territory-wide Clinical Data Analysis and Reporting System (CDARS). The system is an integrative centralized platform that permits the extraction of clinical data for analysis and reporting. The system attributes each patient a unique reference identification number, allowing for the retrieval of comprehensive medical records, including disease diagnoses, clinical comorbidities, laboratory parameters and operative procedures. Patients or the public were not involved in any aspect of this study. The system has been previously used by both our team and other teams in Hong Kong [11–13].

### Patient Data

The following clinical data were extracted: patient characteristics, including demographic details (baseline age and gender), specific pre-existing comorbidities before drug prescriptions, laboratory examinations (including complete blood counts, biochemical tests, lipid/glycemic profiles) were extracted. Past comorbidities from January 1st, 2013, to December 31st, 2020, were extracted, and categorized into hypertension, liver diseases, hip fractures/accident falls, renal diseases, diabetes mellitus, malignant dysrhythmia, chronic obstructive pulmonary disease, ischemic heart disease, peripheral vascular disease, endocrine diseases, gastrointestinal diseases, and stroke/transient ischemic attack. The International Classification of Disease, Ninth Edition (ICD-9) codes that were used to extract the specific comorbidities and outcomes are included in Supplementary Table 2. The overall dosage and the duration of exposure to PD-L1 and PD-1 inhibitors for patients with new-onset cardiac complications are reported.

### Primary outcomes on follow-up

The primary outcome was a composite of incident heart failure, acute myocardial infarction, atrial fibrillation, and atrial flutter. The follow-up period was defined as the first PD-1/PD-L1 prescription date until the primary endpoint or death occurred, or until the end date of August 31st, 2020, whichever was earlier.

### Statistical analysis

Continuous variables were presented as median (95% confidence interval [CI] or interquartile range [IQR]) and categorical variables were presented as count (%). The Mann- Whitney U test was used to compare continuous variables. The χ^2^ test with Yates’ correction was used for 2 × 2 contingency data, and Pearson’s χ^2^ test was used for contingency data for variables with more than two categories. The patients with PD-L1 were matched with PD1 controls through propensity score matching of 1:2 ratio, based on patient demographics, Charlson’s standard comorbidity index, past comorbidities, and non-PD-1/PD-L1 medications. Negligible post-weighting intergroup standardized mean difference (SMD) was defined as SMD < 0.2. To identify the important predictors associated with new-onset cardiac complications of patients after PD-1/PD-L1 treatment, univariable Cox regression was used to calculate hazard ratios (HRs) and 95% CIs. In addition to propensity score matching, the following approaches based on the propensity scores were employed: propensity score stratification [14], inverse probability weighting [15], and high-dimensional propensity score adjustment [16]. Paired hospitalization characteristics of patients before and after treatment were compared both in the unmatched and matched cohorts. A two-sided α of < 0.05 was considered statistically significant. Statistical analyses were performed using RStudio software (Version: 1.1.456) and Python (Version: 3.6).

## Results

### Baseline characteristics

**Fig. 1:**
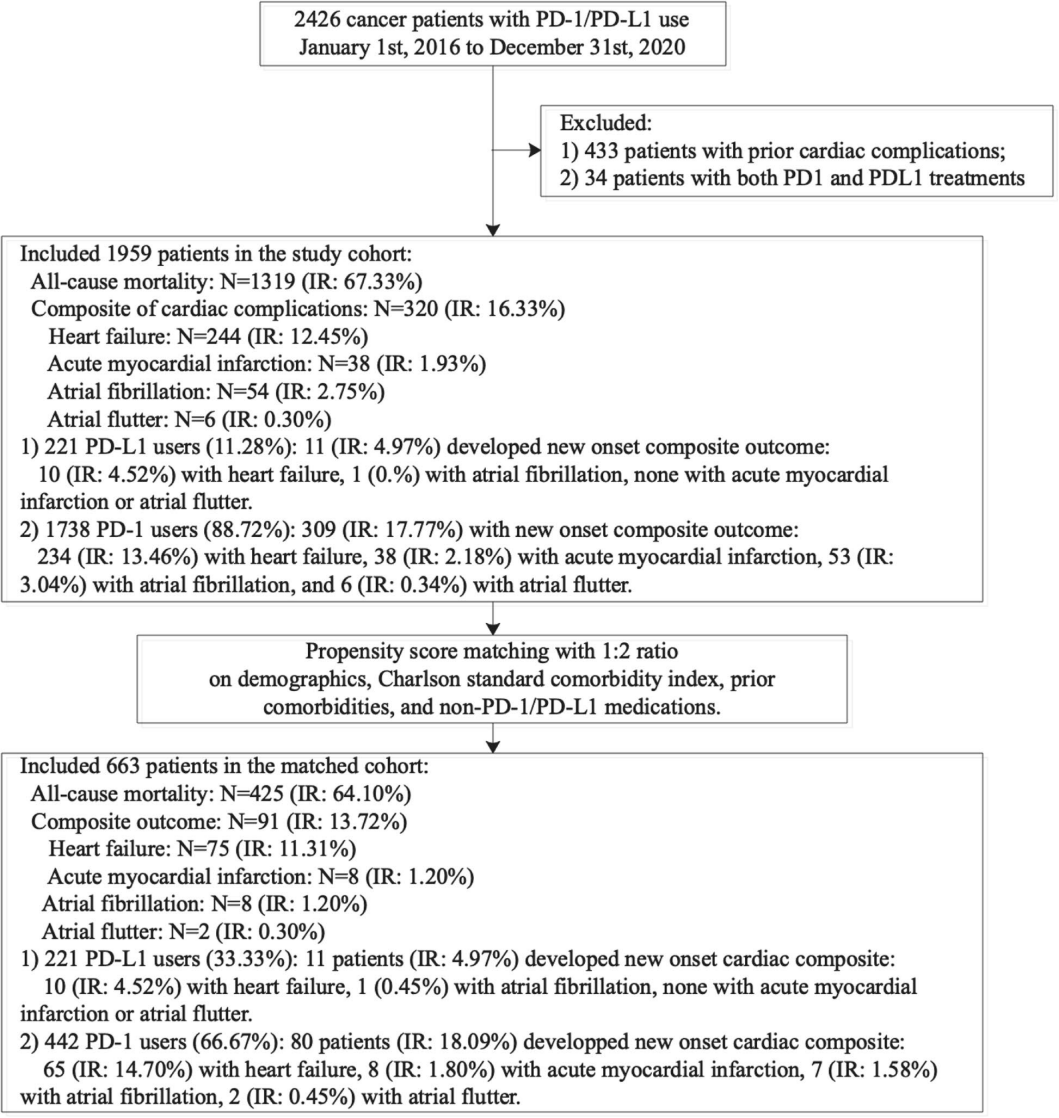
Study flow diagram describing derivation of the study cohort

Initially, 2426 cancer patients receiving PD-1/PD-L1 inhibitors were identified (Fig. 1**)**. In total 1959 patients remained in the study cohort after excluding 433 patients with prior cardiac complications and 34 patients who received both PD1 and PDL1 treatments. Propensity score matching with 1:2 ratio between PD-1 and PD-L1 inhibitor use based on demographics, Charlson’s standard comorbidity index, prior comorbidities, and non-PD-1/PD-L1 medications was performed. This yielded a matched cohort of 663 patients (Table 1).

**Table 1:** Clinical characteristics of patients with PD-1 use and PD-L1 use before and after 1:2 propensity score matching

Characteristics	Before matching				After 1:2 matching			
	All (N = 1959) Mean(SD);N or Count(%)	PD-L1 users (N = 221) Mean(SD);N or Count(%)	PD-1 users (N = 1738) Mean(SD);N or Count(%)	SMD	All (N = 663) Mean(SD);N or Count(%)	PD-L1 users (N = 221) Mean(SD);N or Count(%)	PD-1 users (N = 442) Mean(SD);N or Count(%)	SMD
***Adverse events***								
All-cause mortality	1319(67.33%)	115(52.03%)	1204(69.27%)	0.36^a^	425(64.10%)	115(52.03%)	310(70.13%)	0.38^a^
Composite outcome	320(16.33%)	11(4.97%)	309(17.77%)	0.41^a^	91(13.72%)	11(4.97%)	80(18.09%)	0.42^a^
Heart failure	244(12.45%)	10(4.52%)	234(13.46%)	0.32^a^	75(11.31%)	10(4.52%)	65(14.70%)	0.35^a^
Acute myocardial infarction	38(1.93%)	0(0.00%)	38(2.18%)	0.21^a^	8(1.20%)	0(0.00%)	8(1.80%)	0.19
Atrial fibrillation	54(2.75%)	1(0.45%)	53(3.04%)	0.2	8(1.20%)	1(0.45%)	7(1.58%)	0.11
Atrial flutter	6(0.30%)	0(0.00%)	6(0.34%)	0.08	2(0.30%)	0(0.00%)	2(0.45%)	0.1
***Demographics***								
Male gender	1341(68.45%)	165(74.66%)	1176(67.66%)	0.15	498(75.11%)	165(74.66%)	333(75.33%)	0.02
Female gender	618(31.54%)	56(25.33%)	562(32.33%)	0.15	165(24.88%)	56(25.33%)	109(24.66%)	0.02
Baseline age, years	61.0(13.7);n = 1959	63.1(10.2);n = 221	60.7(14.1);n = 1738	0.19	63.0(10.2);n = 663	63.1(10.2);n = 221	63.0(10.1);n = 442	0
<40	151(7.70%)	7(3.16%)	144(8.28%)	0.22^a^	20(3.01%)	7(3.16%)	13(2.94%)	0.01
[40, 50)	194(9.90%)	13(5.88%)	181(10.41%)	0.17	43(6.48%)	13(5.88%)	30(6.78%)	0.04
[50-60)	481(24.55%)	51(23.07%)	430(24.74%)	0.04	155(23.37%)	51(23.07%)	104(23.52%)	0.01
[60-70)	631(32.21%)	96(43.43%)	535(30.78%)	0.26^a^	288(43.43%)	96(43.43%)	192(43.43%)	0
[70-80)	391(19.95%)	48(21.71%)	343(19.73%)	0.05	133(20.06%)	48(21.71%)	85(19.23%)	0.06
>=80	111(5.66%)	6(2.71%)	105(6.04%)	0.16	24(3.61%)	6(2.71%)	18(4.07%)	0.08
***Past comorbidities***								
Charlson’s standard comorbidity index	6.1(3.3);n = 1959	6.5(3.1);n = 221	6.0(3.3);n = 1738	0.16	6.5(3.1);n = 663	6.53(3.1);n = 221	6.48(3.08);n = 442	0.02
Hypertension	256(13.06%)	29(13.12%)	227(13.06%)	0	88(13.27%)	29(13.12%)	59(13.34%)	0.01
Liver diseases	193(9.85%)	8(3.61%)	185(10.64%)	0.28^a^	24(3.61%)	8(3.61%)	16(3.61%)	0
Hip fractures/accident falls	77(3.93%)	13(5.88%)	64(3.68%)	0.1	37(5.58%)	13(5.88%)	24(5.42%)	0.02
Renal diseases	292(14.90%)	29(13.12%)	263(15.13%)	0.06	85(12.82%)	29(13.12%)	56(12.66%)	0.01
Diabetes mellitus	156(7.96%)	20(9.04%)	136(7.82%)	0.04	60(9.04%)	20(9.04%)	40(9.04%)	0
Malignant dysrhythmia	12(0.61%)	0(0.00%)	12(0.69%)	0.12	2(0.30%)	0(0.00%)	2(0.45%)	0.1
Chronic obstructive pulmonary disease	15(0.76%)	2(0.90%)	13(0.74%)	0.02	6(0.90%)	2(0.90%)	4(0.90%)	0
Ischemic heart disease	61(3.11%)	5(2.26%)	56(3.22%)	0.06	15(2.26%)	5(2.26%)	10(2.26%)	0
Peripheral vascular disease	11(0.56%)	1(0.45%)	10(0.57%)	0.02	3(0.45%)	1(0.45%)	2(0.45%)	0
Endocrine diseases	548(27.97%)	56(25.33%)	492(28.30%)	0.07	166(25.03%)	56(25.33%)	110(24.88%)	0.01
Gastrointestinal diseases	1412(72.07%)	184(83.25%)	1228(70.65%)	0.30^a^	551(83.10%)	184(83.25%)	367(83.03%)	0.01
Stroke/transient ischemic attack	80(4.08%)	8(3.61%)	72(4.14%)	0.03	24(3.61%)	8(3.61%)	16(3.61%)	0
***Hospitalization***								
Average readmission	68.5(194.8);n = 1876	53.3(112.0);n = 215	70.5(203.0);n = 1661	0.1	65.3(202.3);n = 647	53.3(112.0);n = 215	71.3(234.5);n = 432	0.1
Total episode number	14.2(13.9);n = 1876	12.2(8.9);n = 215	14.5(14.4);n = 1661	0.19	13.8(10.9);n = 647	12.2(8.9);n = 215	14.6(11.7);n = 432	0.22^a^
Overall hospital stay, days	35.2(38.2);n = 1876	25.5(22.4);n = 215	36.5(39.6);n = 1661	0.34^a^	32.9(31.3);n = 647	25.5(22.4);n = 215	36.7(34.4);n = 432	0.39^a^
***Medications***								
PD-L1 expenditure, HKD	98251.0(98697.8);n = 221	98251.0(98697.8);n = 221	-	-	98251.0(98697.8);n = 221	98251.0(98697.8);n = 221	-	-
Total PD-L1 dose amount, mg	12644.1(27488.6);n = 221	12644.1(27488.6);n = 221	-	-	12644.1(27488.6);n = 221	12644.1(27488.6);n = 221	-	-
PD-L1 inhibitors duration, days	176.0(200.9);n = 221	176.0(200.9);n = 221	-	-	176.0(200.9);n = 221	176.0(200.9);n = 221	-	-
PD-1 expenditure, HKD	193878.8(291968.7);n = 1750	-	192915.5(291869.7);n = 1738	-	203782.5(259059.2);n = 454	-	200263.3(257787.4);n = 442	-
Total PD-1 dose amount (mg)	2817.9(10492.2);n = 1750	-	2827.3(10527.4);n = 1738	-	2454.7(5152.0);n = 454	-	2482.0(5216.3);n = 442	-
PD-1 inhibitors duration, days	202.7(237.7);n = 1750	-	201.5(237.0);n = 1738	-	207.7(234.7);n = 454	-	202.9(232.1);n = 442	-
Anticoagulants	1108(56.55%)	120(54.29%)	988(56.84%)	0.05	362(54.60%)	120(54.29%)	242(54.75%)	0.01
Steroids	1108(56.55%)	120(54.29%)	988(56.84%)	0.05	362(54.60%)	120(54.29%)	242(54.75%)	0.01
***biomarkers***								
Neutrophil-to-lymphocyte ratio	4.6(6.4);n = 1952	4.5(9.5);n = 221	4.6(5.9);n = 1731	0.01	4.3(6.5);n = 663	4.5(9.5);n = 221	4.2(4.2);n = 442	0.04
Platelet-to-lymphocyte ratio	212.3(243.9);n = 1953	203.9(188.4);n = 221	213.4(250.1);n = 1732	0.04	210.2(304.3);n = 663	203.9(188.4);n = 221	213.3(348.3);n = 442	0.03
Aspartate transaminase-to-alanine transaminase ratio	1.8(3.6);n = 1308	1.1(0.5);n = 128	1.9(3.8);n = 1180	0.29^a^	1.5(2.1);n = 405	1.1(0.5);n = 128	1.7(2.4);n = 277	0.32^a^
Triglyceride glucose index	7.1(0.6);n = 580	7.07(0.65);n = 67	7.06(0.62);n = 513	0.02	7.0(0.6);n = 207	7.1(0.6);n = 67	7.0(0.6);n = 140	0.07
Urea-to-creatinine ratio	73.1(40.8);n = 1937	68.3(23.3);n = 221	73.8(42.4);n = 1716	0.16	69.5(29.2);n = 659	68.3(23.3);n = 221	70.1(31.8);n = 438	0.07
Monocyte-to-lymphocyte ratio	0.4(0.5);n = 1950	0.4(0.5);n = 221	0.5(0.5);n = 1729	0.03	0.4(0.4);n = 662	0.4(0.5);n = 221	0.5(0.4);n = 441	0.04
***Complete blood counts***								
Mean corpuscular volume, fL	88.0(8.2);n = 1953	88.4(7.7);n = 221	88.0(8.2);n = 1732	0.06	88.2(7.7);n = 663	88.4(7.7);n = 221	88.1(7.8);n = 442	0.04
Eosinophil, x10^9/L	0.2(0.3);n = 1952	0.2(0.32);n = 221	0.18(0.31);n = 1731	0.04	0.2(0.3);n = 663	0.2(0.32);n = 221	0.21(0.24);n = 442	0.03
Lymphocyte, x10^9/L	1.5(0.9);n = 1953	1.6(0.8);n = 221	1.5(0.9);n = 1732	0.19	1.5(0.7);n = 663	1.6(0.8);n = 221	1.5(0.7);n = 442	0.21^a^
Metamyelocyte, x10^9/L	0.8(5.0);n = 225	0.5(0.7);n = 13	0.8(5.2);n = 212	0.07	0.3(0.5);n = 72	0.5(0.7);n = 13	0.3(0.4);n = 59	0.37^a^
Monocyte, x10^9/L	0.5(0.3);n = 1953	0.54(0.25);n = 221	0.53(0.32);n = 1732	0.02	0.5(0.3);n = 663	0.54(0.25);n = 221	0.55(0.32);n = 442	0.03
Neutrophil, x10^9/L	5.1(3.3);n = 1953	5.2(3.4);n = 221	5.0(3.3);n = 1732	0.05	5.0(3.2);n = 663	5.2(3.4);n = 221	4.9(3.1);n = 442	0.09
White blood count, x10^9/L	7.4(5.5);n = 1953	7.43(3.3);n = 221	7.39(5.74);n = 1732	0.01	7.3(3.6);n = 663	7.4(3.3);n = 221	7.3(3.8);n = 442	0.04
Mean cell haemoglobin, pg	30.7(3.3);n = 1953	30.72(3.29);n = 221	30.65(3.34);n = 1732	0.02	30.8(3.2);n = 663	30.7(3.3);n = 221	30.8(3.1);n = 442	0.02
Myelocyte, x10^9/L	0.8(3.1);n = 328	1.0(2.0);n = 26	0.7(3.2);n = 302	0.08	0.6(1.5);n = 97	1.0(2.0);n = 26	0.5(1.2);n = 71	0.26^a^
Platelet, x10^9/L	247.9(108.5);n = 1953	261.5(92.6);n = 221	246.2(110.3);n = 1732	0.15	252.6(98.9);n = 663	261.5(92.6);n = 221	248.2(101.7);n = 442	0.14
Red blood count, x10^12/L	4.4(0.7);n = 1953	4.6(0.6);n = 221	4.4(0.7);n = 1732	0.27^a^	4.5(0.7);n = 663	4.6(0.6);n = 221	4.4(0.7);n = 442	0.21^a^
Hematocrit, L/L	0.4(0.1);n = 1895	0.4(0.05);n = 208	0.38(0.06);n = 1687	0.37^a^	0.4(0.1);n = 626	0.4(0.05);n = 208	0.39(0.05);n = 418	0.28^a^
***Renal and liver functions***								
Potassium, mmol/L	4.1(0.4);n = 1941	4.14(0.43);n = 221	4.12(0.44);n = 1720	0.04	4.1(0.4);n = 661	4.14(0.43);n = 221	4.13(0.42);n = 440	0.03
Urate, mmol/L	0.3(0.1);n = 627	0.33(0.1);n = 43	0.33(0.14);n = 584	0.03	0.3(0.1);n = 186	0.33(0.1);n = 43	0.34(0.13);n = 143	0.07
Albumin, g/L	39.2(5.9);n = 1939	39.8(5.4);n = 220	39.1(6.0);n = 1719	0.11	39.5(5.4);n = 660	39.8(5.4);n = 220	39.4(5.4);n = 440	0.06
Sodium, mmol/L	139.1(3.8);n = 1941	139.4(3.1);n = 221	139.1(3.9);n = 1720	0.09	139.2(3.4);n = 661	139.4(3.1);n = 221	139.2(3.6);n = 440	0.07
Urea, mmol/L	5.5(2.4);n = 1937	5.3(1.8);n = 221	5.6(2.5);n = 1716	0.11	5.3(1.8);n = 659	5.31(1.76);n = 221	5.31(1.83);n = 438	0
Protein, g/L	72.1(12.8);n = 1853	71.3(16.3);n = 207	72.2(12.3);n = 1646	0.06	71.7(14.4);n = 624	71.3(16.3);n = 207	71.9(13.5);n = 417	0.04
Bilirubin, umol/L	13.0(25.3);n = 1941	9.5(6.1);n = 221	13.4(26.7);n = 1720	0.2	11.3(19.1);n = 661	9.5(6.1);n = 221	12.2(22.9);n = 440	0.16
Creatinine, umol/L	84.9(62.4);n = 1952	83.0(37.0);n = 221	85.1(64.9);n = 1731	0.04	84.8(64.0);n = 662	83.0(37.0);n = 221	85.7(74.0);n = 441	0.05
SD of creatinine	35.5(103.7);n = 1942	29.8(93.9);n = 220	36.2(104.9);n = 1722	0.06	29.7(82.3);n = 658	29.8(93.9);n = 220	29.7(75.9);n = 438	0
Aspartate transaminase, U/L	54.4(104.9);n = 1371	28.3(21.8);n = 142	57.5(110.1);n = 1229	0.37^a^	41.2(67.2);n = 443	28.3(21.8);n = 142	47.3(79.5);n = 301	0.33^a^
SD of aspartate transaminase	59.6(189.6);n = 1250	28.6(101.7);n = 122	62.9(196.5);n = 1128	0.22^a^	36.5(84.3);n = 409	28.6(101.7);n = 122	39.8(75.6);n = 287	0.13
Alkaline phosphatase, U/L	108.6(111.6);n = 1941	85.2(36.4);n = 221	111.6(117.5);n = 1720	0.3^a^	102.1(99.9);n = 661	85.2(36.4);n = 221	110.5(118.9);n = 440	0.29^a^
SD of alkaline phosphatase	64.8(95.1);n = 1928	39.6(75.3);n = 220	68.0(96.9);n = 1708	0.33^a^	58.3(89.9);n = 655	39.6(75.3);n = 220	67.7(95.2);n = 435	0.33^a^
Alanine transaminase, U/L	36.6(55.7);n = 1876	28.2(25.1);n = 207	37.6(58.3);n = 1669	0.21^a^	31.4(29.5);n = 623	28.2(25.1);n = 207	33.1(31.4);n = 416	0.17
SD of alanine transaminase	36.4(84.7);n = 1855	22.9(50.7);n = 201	38.0(87.8);n = 1654	0.21^a^	28.2(51.6);n = 612	22.9(50.7);n = 201	30.8(52.0);n = 411	0.15
***Lipid, iron and calcium profile***								
Total iron-binding capacity, L	41.0(12.3);n = 103	39.1(11.1);n = 5	41.1(12.4);n = 98	0.16	42.1(12.7);n = 22	39.1(11.1);n = 5	43.0(13.3);n = 17	0.31^a^
VitaminB12, pmol/L	453.2(327.2);n = 49	447.7(265.6);n = 3	453.6(333.2);n = 46	0.02	401.2(305.7);n = 17	447.7(265.6);n = 3	391.2(321.8);n = 14	0.19
Folate, ng/mL	21.7(10.2);n = 69	16.0(3.7);n = 3	22.0(10.3);n = 66	0.78^a^	20.4(10.4);n = 19	16.0(3.7);n = 3	21.3(11.1);n = 16	0.64^a^
Ferritin, pmol/L	2546.7(4740.6);n = 86	728.2(816.5);n = 8	2733.2(4936.4);n = 78	0.57^a^	1941.1(2987.1);n = 22	728.2(816.5);n = 8	2634.2(3554.1);n = 14	0.74^a^
Calcium, mmol/L	2.3(0.2);n = 1081	2.32(0.13);n = 82	2.32(0.16);n = 999	0.04	2.3(0.1);n = 340	2.32(0.13);n = 82	2.33(0.15);n = 258	0.04
SD of calcium	0.1(0.1);n = 987	0.07(0.04);n = 73	0.1(0.06);n = 914	0.49^a^	0.1(0.1);n = 314	0.07(0.04);n = 73	0.1(0.07);n = 241	0.43^a^
Phosphate, mmol/L	1.1(0.2);n = 948	1.08(0.18);n = 61	1.06(0.22);n = 887	0.11	1.1(0.2);n = 286	1.08(0.18);n = 61	1.06(0.2);n = 225	0.1
SD of phosphate	0.2(0.1);n = 785	0.1(0.1);n = 44	0.2(0.1);n = 741	0.41^a^	0.2(0.1);n = 241	0.1(0.1);n = 44	0.2(0.1);n = 197	0.37^a^
***Glycemic and clotting profile***								
Triglyceride, mmol/L	1.4(1.1);n = 598	1.5(1.0);n = 68	1.4(1.1);n = 530	0.08	1.5(1.2);n = 214	1.5(1.05);n = 68	1.46(1.29);n = 146	0.04
SD of triglyceride	0.4(0.5);n = 294	0.4(0.43);n = 30	0.35(0.54);n = 264	0.1	0.4(0.6);n = 90	0.4(0.43);n = 30	0.42(0.66);n = 60	0.04
HbA1c, g/dL	6.3(2.3);n = 1773	6.4(2.7);n = 206	6.2(2.2);n = 1567	0.09	6.3(2.2);n = 608	6.4(2.7);n = 206	6.2(2.0);n = 402	0.11
SD of HbA1c, g/dL	1.5(1.3);n = 1638	1.4(1.3);n = 194	1.5(1.3);n = 1444	0.09	1.5(1.4);n = 564	1.4(1.3);n = 194	1.6(1.4);n = 370	0.15
Glucose, mmol/L	12.8(2.0);n = 1953	13.3(1.7);n = 221	12.8(2.0);n = 1732	0.31^a^	13.1(1.9);n = 663	13.3(1.7);n = 221	12.9(1.9);n = 442	0.23^a^
SD of glucose, mmol/L	1.3(0.5);n = 1947	1.31(0.54);n = 220	1.29(0.54);n = 1727	0.05	1.3(0.5);n = 660	1.31(0.54);n = 220	1.32(0.51);n = 440	0.03
High sensitive troponin-I, ng/L	363.3(10967.7);n = 1141	12.7(31.8);n = 122	405.3(11605.6);n = 1019	0.05	35.5(314.8);n = 372	12.7(31.8);n = 122	46.6(383.1);n = 250	0.12
SD of high sensitive troponin-I	124.0(1328.2);n = 755	18.2(42.4);n = 82	136.9(1406.3);n = 673	0.12	34.7(207.0);n = 245	18.2(42.4);n = 82	43.0(251.9);n = 163	0.14
APTT, second	30.8(5.5);n = 694	29.1(3.5);n = 46	30.9(5.6);n = 648	0.39^a^	30.4(3.9);n = 217	29.1(3.5);n = 46	30.8(3.9);n = 171	0.45^a^
SD of APTT	2.6(3.1);n = 510	1.9(2.3);n = 26	2.6(3.2);n = 484	0.28^a^	2.1(1.8);n = 152	1.9(2.3);n = 26	2.1(1.7);n = 126	0.13
Lactate dehydrogenase, U/L	324.1(422.0);n = 1431	315.6(534.6);n = 145	325.1(407.6);n = 1286	0.02	302.1(354.0);n = 466	315.6(534.6);n = 145	296.0(231.0);n = 321	0.05
SD of lactate dehydrogenase	155.2(397.1);n = 1140	115.6(203.9);n = 102	159.1(411.1);n = 1038	0.13	121.3(206.3);n = 365	115.6(203.9);n = 102	123.6(207.6);n = 263	0.04
Total cholesterol, mmol/L	4.6(1.1);n = 599	4.9(1.0);n = 68	4.5(1.1);n = 531	0.29^a^	4.6(1.1);n = 214	4.9(1.0);n = 68	4.5(1.1);n = 146	0.3^a^
SD of total cholesterol	0.5(0.4);n = 295	0.54(0.43);n = 31	0.48(0.44);n = 264	0.14	0.5(0.5);n = 93	0.54(0.43);n = 31	0.48(0.52);n = 62	0.13
Low-density lipoprotein, mmol/L	2.6(0.9);n = 583	2.9(0.9);n = 65	2.6(0.9);n = 518	0.34^a^	2.7(1.0);n = 208	2.9(0.9);n = 65	2.6(1.0);n = 143	0.33^a^
SD of low-density lipoprotein	0.4(0.4);n = 283	0.5(0.4);n = 29	0.4(0.4);n = 254	0.18	0.4(0.4);n = 88	0.5(0.4);n = 29	0.4(0.4);n = 59	0.21^a^
High-density lipoprotein, mmol/L	1.3(0.4);n = 593	1.32(0.4);n = 67	1.34(0.4);n = 526	0.04	1.3(0.4);n = 212	1.32(0.4);n = 67	1.31(0.36);n = 145	0.02
SD of high-density lipoprotein	0.1(0.1);n = 280	0.12(0.09);n = 30	0.14(0.14);n = 250	0.17	0.2(0.1);n = 87	0.1(0.1);n = 30	0.2(0.2);n = 57	0.32^a^

^a^ for SMD≥0.2; SD: Standard deviation; SMD: Standard mean difference; APTT: applied partial thromboplastin test; PD-1: Programmed death 1 inhibitors; PD-L1: programmed death 1 ligand inhibitors

Propensity score matching in 1:2 ratio between PD-1 users and PD-L1 users using the nearest neighbor search strategy (caliper as 0.1) was used. The results of logistics regression for potential confounders used in propensity score calculations, balance between groups, and estimations of bootstrapped standard error are shown in Supplementary Tables 3, 4 and 5, respectively. Distributions of propensity scores before and after matching are shown in Supplementary Fig. 1. These results indicate that the covariables between the groups are balanced after matching.

### Adverse cardiovascular outcomes on follow-up and their significant predictors

In the matched cohort, 425 (IR: 64.10%) patients died and 91 (IR: 13.72%) developed new onset cardiac composite outcome. Amongst the latter, 75 (IR: 11.31%) developed heart failure, 8 (IR: 1.20%) developed acute myocardial infarction, 8 (IR: 1.20%) developed atrial fibrillation and 2 (IR: 0.30%) developed atrial flutter. The incidence rate of the composite outcome was lower in the PD-L1 cohort than in the PD-1 cohort (7.0% vs. 20.7%; P < 0.001).

In the 221 PD-L1 users, there were 11 patients (IR: 4.97%) who developed the composite outcome, in which 10 (IR: 4.52%) with heart failure, 1 (0.45%) with atrial fibrillation, but none with acute myocardial infarction or atrial flutter. In the 442 patients PD-1 users, 80 patients (IR: 18.09%) developed the composite outcome. Of the latter group, 65 (IR: 14.70%) developed heart failure, 8 (IR: 1.80%) developed acute myocardial infarction, 7 (IR: 1.58%) developed atrial fibrillation, and 2 (IR: 0.45%) developed atrial flutter.

**Fig. 2:**
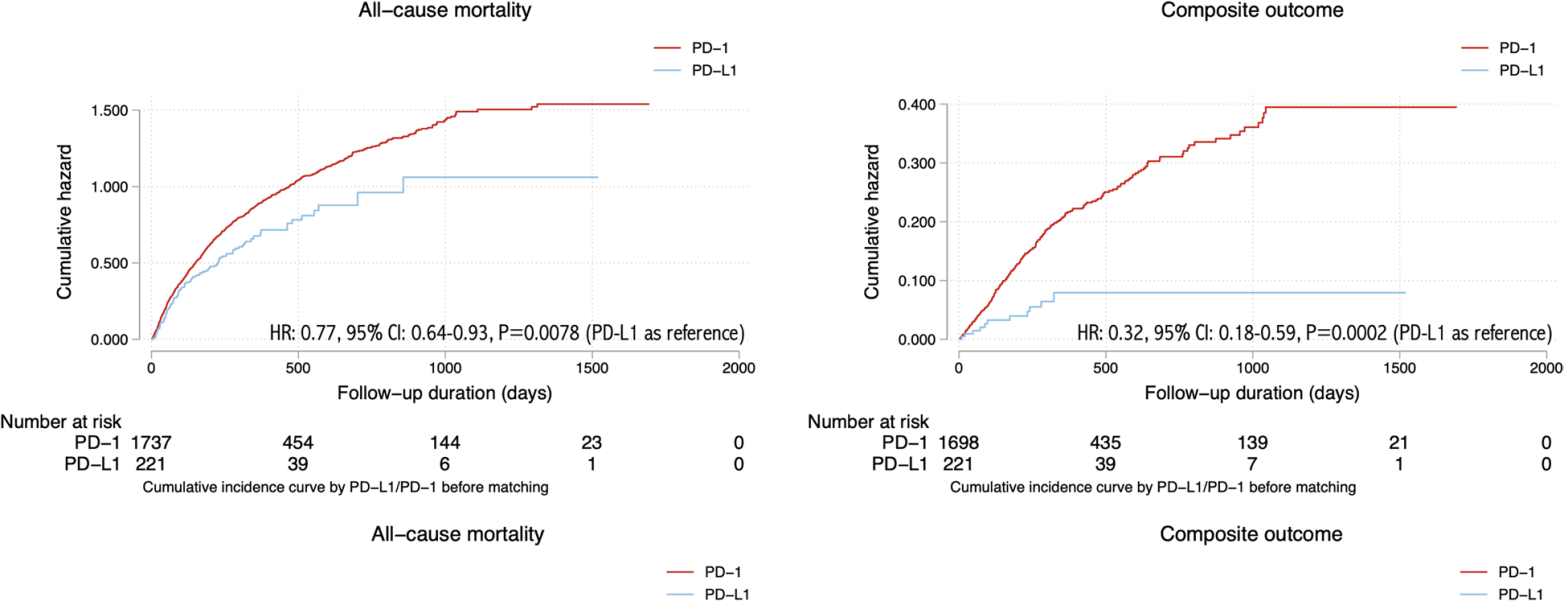
Cumulative incidence curves of new onset cardiac complications and all-cause mortality in cancer patients stratified by PD-1 or PD-L1 inhibitor use before 1:2 propensity score matching

**Fig. 3:**
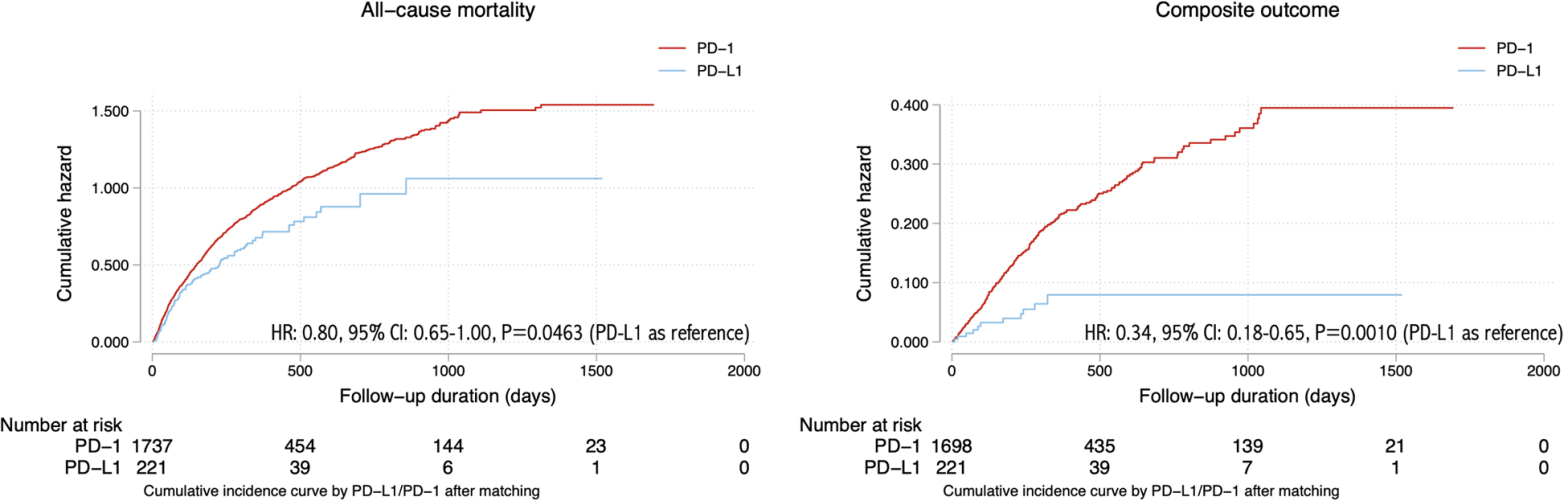
Cumulative incidence curves of new onset cardiac complications and all-cause mortality in cancer patients stratified by PD-1 or PD-L1 inhibitor use after 1:2 propensity score matching

The breakdown on the individual adverse events is shown in Fig. 1 and the patient characteristics stratified by adverse cardiovascular outcomes are shown in Tables 2 and 3. The cumulative incidence curves of new onset cardiac complications and all-cause mortality in cancer patients stratified by PD-1 or PD-L1 inhibitor use before and after 1:2 propensity score matching were presented in Figs. 2 and 3, respectively. The baseline characteristics of the cohort stratified by mortality status are shown in Supplementary Table 6.

**Table 2:** Clinical characteristics of patients who developed the composite outcome before and after 1:2 propensity score matching

Characteristics	Before matching		SMD	After 1:2 matching		SMD
	Composite outcome (N = 320) Mean(SD);N or Count(%)	No composite outcome (N = 1639) Mean(SD);N or Count(%)		Composite outcome (N = 91) Mean(SD);N or Count(%)	No composite outcome (N = 572) Mean(SD);N or Count(%)	
***Demographics***						
Male gender	219(68.43%)	1122(68.45%)	<0.01	69(75.82%)	429(75.00%)	0.02
Female gender	101(31.56%)	517(31.54%)	<0.01	22(24.17%)	143(25.00%)	0.02
Baseline age, years	64.0(12.8);n = 320	60.4(13.8);n = 1639	0.27^a^	62.7(10.3);n = 91	63.1(10.1);n = 572	0.04
<40	16(5.00%)	135(8.23%)	0.13	4(4.39%)	16(2.79%)	0.09
[40, 50)	24(7.50%)	170(10.37%)	0.1	6(6.59%)	37(6.46%)	0.01
[50-60)	70(21.87%)	411(25.07%)	0.08	24(26.37%)	131(22.90%)	0.08
[60-70)	106(33.12%)	525(32.03%)	0.02	36(39.56%)	252(44.05%)	0.09
[70-80)	74(23.12%)	317(19.34%)	0.09	17(18.68%)	116(20.27%)	0.04
>=80	30(9.37%)	81(4.94%)	0.17	4(4.39%)	20(3.49%)	0.05
***Past comorbidities***						
Charlson’s standard comorbidity index	6.7(3.3);n = 320	6.0(3.3);n = 1639	0.23^a^	7.0(3.1);n = 91	6.4(3.1);n = 572	0.18
Hypertension	49(15.31%)	207(12.62%)	0.08	13(14.28%)	75(13.11%)	0.03
Liver diseases	22(6.87%)	171(10.43%)	0.13	3(3.29%)	21(3.67%)	0.02
Hip fractures/accident falls	19(5.93%)	58(3.53%)	0.11	9(9.89%)	28(4.89%)	0.19
Renal diseases	55(17.18%)	237(14.46%)	0.07	12(13.18%)	73(12.76%)	0.01
Diabetes mellitus	30(9.37%)	126(7.68%)	0.06	10(10.98%)	50(8.74%)	0.08
Malignant dysrhythmia	10(3.12%)	2(0.12%)	0.24^a^	0(0.00%)	2(0.34%)	0.08
Chronic obstructive pulmonary disease	3(0.93%)	12(0.73%)	0.02	2(2.19%)	4(0.69%)	0.13
Ischemic heart disease	19(5.93%)	42(2.56%)	0.17	3(3.29%)	12(2.09%)	0.07
Peripheral vascular disease	3(0.93%)	8(0.48%)	0.05	1(1.09%)	2(0.34%)	0.09
Endocrine diseases	76(23.75%)	472(28.79%)	0.11	20(21.97%)	146(25.52%)	0.08
Gastrointestinal diseases	230(71.87%)	1182(72.11%)	0.01	82(90.10%)	469(81.99%)	0.24^a^
Stroke/transient ischemic attack	17(5.31%)	63(3.84%)	0.07	3(3.29%)	21(3.67%)	0.02
***Hospitalization***						
Average readmission	45.2(87.1);n = 312	73.1(209.5);n = 1564	0.17	31.0(35.6);n = 90	70.9(217.1);n = 557	0.26^a^
Total episode number	13.2(11.4);n = 312	14.4(14.4);n = 1564	0.09	14.5(9.0);n = 90	13.7(11.1);n = 557	0.08
Overall hospital stay, days	40.4(38.3);n = 312	34.2(38.1);n = 1564	0.16	44.7(34.7);n = 90	31.0(30.4);n = 557	0.42^a^
***Medications***						
PD-L1 v.s. PD-1	11(3.43%)	210(12.81%)	0.35^a^	11(12.08%)	210(36.71%)	0.60^a^
PD-L1 expenditure, HKD	58752.6(47219.8);n = 11	100319.9(100303.8);n = 210	0.53^a^	58752.6(47219.8);n = 11	100319.9(100303.8);n = 210	0.53^a^
Total PD-L1 dose amount, mg	5127.3(4650.4);n = 11	13037.9(28128.8);n = 210	0.39^a^	5127.3(4650.4);n = 11	13037.9(28128.8);n = 210	0.39^a^
PD-L1 inhibitors duration, days	84.6(114.7);n = 11	180.7(203.5);n = 210	0.58^a^	84.6(114.7);n = 11	180.7(203.5);n = 210	0.58^a^
PD-1 expenditure, HKD	163440.0(284113.0);n = 309	200406.0(293311.2);n = 1441	0.13	183754.8(224766.1);n = 80	208066.4(265897.1);n = 374	0.1
Total PD-1 dose amount (mg)	5146.2(21240.1);n = 309	2318.6(5983.5);n = 1441	0.18	3948.7(9243.4);n = 80	2135.1(3682.3);n = 374	0.26^a^
PD-1 inhibitors duration, days	172.1(197.1);n = 309	209.3(245.1);n = 1441	0.17	173.9(197.7);n = 80	214.9(241.5);n = 374	0.19
Anticoagulants	171(53.43%)	937(57.16%)	0.08	47(51.64%)	315(55.06%)	0.07
Steroids	171(53.43%)	937(57.16%)	0.08	47(51.64%)	315(55.06%)	0.07
***Biomarkers***						
Neutrophil-to-lymphocyte ratio	4.3(6.8);n = 320	4.6(6.3);n = 1632	0.05	3.5(1.8);n = 91	4.4(6.9);n = 572	0.19
Platelet-to-lymphocyte ratio	209.3(237.5);n = 320	212.9(245.2);n = 1633	0.02	199.9(135.5);n = 91	211.8(323.2);n = 572	0.05
Aspartate transaminase-to-alanine transaminase ratio	1.7(1.7);n = 204	1.8(3.9);n = 1104	0.05	1.2(0.6);n = 52	1.5(2.2);n = 353	0.2
Triglyceride glucose index	6.9(0.5);n = 106	7.1(0.6);n = 474	0.35^a^	6.8(0.5);n = 28	7.1(0.6);n = 179	0.44^a^
Urea-to-creatinine ratio	72.9(30.7);n = 317	73.2(42.5);n = 1620	0.01	78.0(45.9);n = 91	68.1(25.3);n = 568	0.26^a^
Monocyte-to-lymphocyte ratio	0.4(0.4);n = 319	0.5(0.5);n = 1631	0.05	0.44(0.36);n = 90	0.45(0.45);n = 572	0.03
***Complete blood counts***						
Hb, g/dL	12.9(1.9);n = 320	12.8(2.0);n = 1633	0.03	13.6(1.5);n = 91	13.0(1.9);n = 572	0.35^a^
SD of Hb	1.4(0.5);n = 320	1.3(0.6);n = 1627	0.14	1.5(0.4);n = 91	1.3(0.5);n = 569	0.41^a^
Mean corpuscular volume, fL	88.9(7.8);n = 320	87.8(8.2);n = 1633	0.13	89.9(5.1);n = 91	87.9(8.0);n = 572	0.29^a^
Eosinophil, x10^9/L	0.18(0.2);n = 320	0.19(0.33);n = 1632	0.02	0.21(0.21);n = 91	0.2(0.27);n = 572	0.05
Lymphocyte, x10^9/L	1.6(1.0);n = 320	1.5(0.9);n = 1633	0.08	1.6(0.6);n = 91	1.5(0.7);n = 572	0.09
Metamyelocyte, x10^9/L	0.3(0.6);n = 37	0.9(5.5);n = 188	0.13	0.35(0.36);n = 11	0.34(0.53);n = 61	0.02
Monocyte, x10^9/L	0.54(0.3);n = 320	0.53(0.31);n = 1633	0.02	0.6(0.4);n = 91	0.5(0.3);n = 572	0.1
Neutrophil, x10^9/L	5.0(3.2);n = 320	5.1(3.3);n = 1633	0.02	4.99(2.76);n = 91	5.02(3.28);n = 572	0.01
White blood count, x10^9/L	7.41(3.71);n = 320	7.39(5.8);n = 1633	0	7.4(3.2);n = 91	7.3(3.7);n = 572	0.01
Mean cell haemoglobin, pg	30.9(3.2);n = 320	30.6(3.4);n = 1633	0.08	31.4(2.4);n = 91	30.7(3.3);n = 572	0.24^a^
Myelocyte, x10^9/L	0.5(0.9);n = 50	0.8(3.3);n = 278	0.14	0.4(0.5);n = 16	0.7(1.6);n = 81	0.27^a^
Platelet, x10^9/L	248.7(100.8);n = 320	247.8(110.0);n = 1633	0.01	258.9(81.8);n = 91	251.6(101.4);n = 572	0.08
Red blood count, x10^12/L	4.36(0.68);n = 320	4.4(0.71);n = 1633	0.06	4.54(0.51);n = 91	4.45(0.68);n = 572	0.14
Hematocrit, L/L	0.38(0.05);n = 303	0.38(0.06);n = 1592	0.02	0.41(0.04);n = 79	0.39(0.05);n = 547	0.39^a^
***Renal and liver functions***						
Potassium, mmol/L	4.14(0.45);n = 318	4.12(0.43);n = 1623	0.03	4.12(0.39);n = 91	4.13(0.43);n = 570	0.02
Urate, mmol/L	0.34(0.17);n = 96	0.32(0.13);n = 531	0.14	0.33(0.19);n = 32	0.34(0.11);n = 154	0.02
Albumin, g/L	39.17(5.99);n = 318	39.2(5.89);n = 1621	0	40.4(4.3);n = 91	39.4(5.6);n = 569	0.21^a^
Sodium, mmol/L	139.0(3.8);n = 318	139.1(3.8);n = 1623	0.03	139.2(3.6);n = 91	139.3(3.4);n = 570	0.01
Urea, mmol/L	5.7(2.8);n = 317	5.5(2.4);n = 1620	0.08	5.34(2.09);n = 91	5.3(1.76);n = 568	0.02
Protein, g/L	72.6(12.5);n = 308	72.0(12.9);n = 1545	0.04	72.2(12.5);n = 87	71.6(14.7);n = 537	0.05
Bilirubin, umol/L	11.9(16.6);n = 318	13.2(26.6);n = 1623	0.05	10.9(7.3);n = 91	11.4(20.3);n = 570	0.04
Creatinine, umol/L	84.6(44.3);n = 320	85.0(65.3);n = 1632	0.01	78.0(42.3);n = 91	85.9(66.8);n = 571	0.14
SD of creatinine	39.6(128.3);n = 319	34.7(98.1);n = 1623	0.04	28.8(55.2);n = 91	29.9(85.9);n = 567	0.01
Aspartate transaminase, U/L	45.2(56.6);n = 221	56.2(111.7);n = 1150	0.12	29.0(15.0);n = 64	43.3(72.3);n = 379	0.27^a^
SD of aspartate transaminase	57.8(157.0);n = 198	59.9(195.2);n = 1052	0.01	30.5(74.7);n = 62	37.5(85.9);n = 347	0.09
Alkaline phosphatase, U/L	98.9(76.8);n = 318	110.5(117.1);n = 1623	0.12	87.6(42.5);n = 91	104.4(106.1);n = 570	0.21^a^
SD of alkaline phosphatase	68.8(96.7);n = 317	64.0(94.8);n = 1611	0.05	62.9(90.6);n = 90	57.5(89.9);n = 565	0.06
Alanine transaminase, U/L	36.0(69.2);n = 301	36.7(52.8);n = 1575	0.01	26.7(16.3);n = 79	32.1(30.9);n = 544	0.22^a^
SD of alanine transaminase	40.6(122.4);n = 300	35.6(75.3);n = 1555	0.05	21.6(21.1);n = 78	29.2(54.6);n = 534	0.18
***Lipid, iron and calcium profile***						
Total iron-binding capacity, L	41.4(14.2);n = 13	40.9(12.1);n = 90	0.04	61.3(15.3);n = 2	40.2(11.1);n = 20	1.58^a^
VitaminB12, pmol/L	305.1(109.5);n = 6	473.9(342.5);n = 43	0.66^a^	304.0(145.7);n = 2	414.1(322.1);n = 15	0.44^a^
Folate, ng/mL	26.5(14.2);n = 8	21.1(9.5);n = 61	0.44^a^	32.3(29.6);n = 2	19.0(6.8);n = 17	0.62^a^
Ferritin, pmol/L	3257.7(4708.7);n = 12	2431.4(4767.6);n = 74	0.17	5116.7(6053.8);n = 2	1623.6(2601.0);n = 20	0.75^a^
Calcium, mmol/L	2.31(0.14);n = 185	2.32(0.16);n = 896	0.06	2.34(0.1);n = 53	2.32(0.15);n = 287	0.1
SD of calcium	0.09(0.06);n = 167	0.1(0.06);n = 820	0.05	0.09(0.06);n = 48	0.09(0.07);n = 266	0.01
Phosphate, mmol/L	1.0(0.2);n = 159	1.1(0.2);n = 789	0.05	1.0(0.2);n = 41	1.1(0.2);n = 245	0.09
SD of phosphate	0.17(0.12);n = 122	0.16(0.11);n = 663	0.05	0.17(0.12);n = 33	0.15(0.09);n = 208	0.16
***Glycemic and clotting profile***						
Triglyceride, mmol/L	1.2(0.8);n = 110	1.5(1.1);n = 488	0.24^a^	1.1(0.7);n = 30	1.5(1.3);n = 184	0.38^a^
SD of triglyceride	0.3(0.4);n = 46	0.4(0.5);n = 248	0.23^a^	0.3(0.2);n = 8	0.4(0.6);n = 82	0.28^a^
Glucose, mmol/L	6.2(2.1);n = 294	6.3(2.3);n = 1479	0.03	6.1(1.5);n = 86	6.3(2.3);n = 522	0.13
SD of glucose	1.55(1.26);n = 276	1.48(1.3);n = 1362	0.05	1.6(1.4);n = 83	1.5(1.4);n = 481	0.1
High sensitive troponin-I, ng/L	1660.7(24305.4);n = 232	32.2(442.8);n = 909	0.09	106.2(718.1);n = 70	19.1(50.2);n = 302	0.17
SD of high sensitive troponin-I	367.5(2445.0);n = 164	56.5(762.9);n = 591	0.17	101.3(453.4);n = 48	18.5(51.9);n = 197	0.26^a^
APTT, second	30.4(4.2);n = 118	30.9(5.8);n = 576	0.09	30.36(3.19);n = 34	30.44(3.98);n = 183	0.02
SD of APTT	2.1(1.6);n = 79	2.7(3.3);n = 431	0.24^a^	2.0(1.7);n = 22	2.1(1.8);n = 130	0.02
Lactate dehydrogenase, U/L	326.8(437.7);n = 247	323.6(418.8);n = 1184	0.01	273.8(128.9);n = 72	307.3(380.9);n = 394	0.12
SD of lactate dehydrogenase	133.2(228.0);n = 182	159.3(421.6);n = 958	0.08	131.0(187.3);n = 57	119.5(209.9);n = 308	0.06
Total cholesterol, mmol/L	4.4(1.0);n = 110	4.6(1.1);n = 489	0.18	4.9(1.4);n = 30	4.6(1.0);n = 184	0.24^a^
SD of total cholesterol	0.4(0.4);n = 46	0.5(0.4);n = 249	0.15	0.7(0.9);n = 8	0.5(0.4);n = 85	0.32^a^
Low-density lipoprotein, mmol/L	2.5(0.9);n = 109	2.6(0.9);n = 474	0.13	3.0(1.3);n = 30	2.6(0.9);n = 178	0.3^a^
SD of low-density lipoprotein	0.38(0.35);n = 44	0.42(0.37);n = 239	0.11	0.7(0.8);n = 8	0.4(0.3);n = 80	0.45^a^
High-density lipoprotein, mmol/L	1.4(0.4);n = 109	1.3(0.4);n = 484	0.07	1.4(0.3);n = 30	1.3(0.4);n = 182	0.26^a^
SD of high-density lipoprotein	0.14(0.13);n = 42	0.14(0.13);n = 238	0.02	0.1(0.1);n = 8	0.2(0.1);n = 79	0.02

^a^ for SMD≥0.2; SD: Standard deviation; SMD: Standard mean difference; APTT: applied partial thromboplastin test; PD-1: Programmed death 1 inhibitors; PD-L1: programmed death 1 ligand inhibitors

# indicates the difference between patients with/without the composite outcome

**Table 3:** Significant univariable predictors of new onset cardiac complication outcome and all-cause mortality before and after 1:2 propensity score matching

Characteristics	Before matching		After 1:2 matching	
	All-cause mortality HR [95% CI];P value	Composite outcome HR [95% CI];P value	All-cause mortality HR [95% CI];P value	Composite outcome HR [95% CI];P value
***Demographics***				
Male gender	0.98[0.87-1.10];0.6878	1.00[0.78-1.29];0.9898	1.03[0.83-1.29];0.7733	1.12[0.67-1.88];0.6546
Female gender	1.0[Reference]	1.0[Reference]	1.0[Reference]	1.0[Reference]
Baseline age, years	1.00[1.00-1.01];0.1533	1.01[1.01-1.02];0.0016**	1.01[1.00-1.01];0.2976	1.00[0.98-1.02];0.9421
<40	1.0[Reference]	1.0[Reference]	1.0[Reference]	1.0[Reference]
[40, 50)	1.02[0.85-1.22];0.8400	0.72[0.46-1.12];0.1450	1.07[0.73-1.56];0.7308	0.96[0.39-2.38];0.9321
[50-60)	1.04[0.92-1.18];0.5569	0.97[0.74-1.28];0.8386	0.87[0.69-1.09];0.2345	1.20[0.74-1.95];0.4528
[60-70)	1.02[0.91-1.14];0.7455	1.05[0.82-1.35];0.6880	0.98[0.81-1.19];0.8684	0.71[0.45-1.11];0.1309
[70-80)	0.98[0.85-1.12];0.7150	1.19[0.91-1.57];0.2103	1.16[0.92-1.47];0.2172	1.13[0.66-1.96];0.6528
>=80	1.07[0.86-1.35];0.5366	1.40[0.89-2.21];0.1436	1.03[0.63-1.70];0.9074	1.51[0.55-4.13];0.4210
***Past comorbidities***				
Charlson’s standard comorbidity index	1.05[1.04-1.07];<0.0001***	1.08[1.04-1.12];<0.0001***	1.08[1.04-1.11];<0.0001***	1.07[1.00-1.15];0.0589
Hypertension	1.20[1.02-1.40];0.0262*	1.11[0.78-1.59];0.5645	1.32[1.00-1.73];0.0481*	1.18[0.61-2.29];0.6236
Liver diseases	1.24[1.04-1.47];0.0163*	0.77[0.49-1.22];0.2689	1.46[0.91-2.35];0.1148	1.32[0.42-4.18];0.6381
Hip fractures/accident falls	1.26[0.96-1.64];0.0910	1.98[1.21-3.23];0.0065**	0.79[0.50-1.25];0.3174	2.21[1.10-4.41];0.0253*
Renal diseases	1.02[0.88-1.19];0.7670	1.06[0.77-1.48];0.7155	1.05[0.79-1.39];0.7571	1.24[0.67-2.29];0.4871
Diabetes mellitus	1.13[0.92-1.37];0.2369	1.05[0.67-1.66];0.8204	1.12[0.80-1.57];0.4963	1.12[0.52-2.43];0.7733
Malignant dysrhythmia	2.09[1.12-3.89];0.0206*	11.11[5.88-20.98];<0.0001***	0.00[0.00-Inf];0.9877	0.00[0.00-Inf];0.9946
Chronic obstructive pulmonary disease	1.42[0.78-2.56];0.2515	1.83[0.59-5.72];0.2971	2.04[0.84-4.94];0.1133	5.51[1.34-22.64];0.0179*
Ischemic heart disease	0.98[0.72-1.34];0.8984	1.30[0.69-2.44];0.4160	0.98[0.49-1.98];0.9639	0.70[0.10-5.07];0.7271
Peripheral vascular disease	1.10[0.57-2.12];0.7774	1.93[0.62-6.03];0.2570	0.87[0.22-3.51];0.8502	2.51[0.35-18.07];0.3611
Endocrine diseases	1.04[0.93-1.18];0.4907	0.76[0.58-1.00];0.0538	1.04[0.84-1.29];0.7304	0.75[0.44-1.28];0.2934
Gastrointestinal diseases	1.03[0.92-1.17];0.5810	0.94[0.73-1.21];0.6327	1.22[0.93-1.59];0.1534	1.71[0.85-3.41];0.1296
Stroke/transient ischemic attack	1.11[0.85-1.46];0.4279	1.02[0.54-1.92];0.9477	1.48[0.91-2.40];0.1149	0.96[0.24-3.92];0.9586
***Hospitalization***				
Average readmission	1.000[1.000-1.000];0.2724	0.998[0.997-1.000];0.0625	1.000[1.000-1.001];0.0823	0.99[0.98-1.00];0.0080**
Total episode number	0.95[0.95-0.96];<0.0001***	0.96[0.95-0.97];<0.0001***	0.96[0.95-0.97];<0.0001***	0.97[0.95-0.99];0.0050**
Overall hospital stay, days	1.001[0.999-1.002];0.3779	1.001[0.999-1.004];0.3117	1.00[1.00-1.01];0.0004***	1.01[1.00-1.01];0.0040**
***Medications***				
PD-L1 v.s. PD-1	0.77[0.64-0.93];0.0078**	0.32[0.18-0.59];0.0002***	0.80[0.65-1.00];0.0463*	0.34[0.18-0.65];0.0010**
PD-L1 expenditure, HKD	1.000[1.000-1.0001];<0.0001***	1.000[1.000-1.000];0.0528	1.000[1.000-1.0001];<0.0001***	1.000[1.000-1.000];0.0528
Total PD-L1 dose amount, mg	1.000[1.000-1.000];0.0703	1.000[1.000-1.000];0.2410	1.000[1.000-1.000];0.0703	1.000[1.000-1.000];0.2410
PD-L1 inhibitors duration, days	1.00[0.99-1.001];<0.0001***	0.99[0.99-1.00];0.0263*	1.00[0.99-1.00];<0.0001***	0.99[0.99-1.00];0.0263*
PD-1 expenditure, HKD	1.000[1.000-1.0001];<0.0001***	1.000[1.000-1.0001];<0.0001***	1.000[1.000-1.0001];<0.0001***	1.000[1.000-1.001];0.0018**
Total PD-1 dose amount (mg)	1.000[1.000-1.0001];<0.0001***	1.000[1.000-1.000];0.1842	1.000[1.000-1.0001];0.0008***	1.000[1.000-1.000];0.0958
PD-1 inhibitors duration, days	0.997[0.997-0.998];<0.0001***	0.998[0.997-0.998];<0.0001***	0.998[0.997-0.998];<0.0001***	0.998[0.997-0.999];0.0007***
Anticoagulants	0.81[0.73-0.91];0.0002***	0.73[0.58-0.93];0.0097**	0.89[0.74-1.08];0.2423	0.78[0.50-1.20];0.2574
Steroids	0.81[0.73-0.91];0.0002***	0.73[0.58-0.93];0.0097**	0.89[0.74-1.08];0.2423	0.78[0.50-1.20];0.2574
***Biomarkers***				
Neutrophil-to-lymphocyte ratio	1.01[1.00-1.01];0.0296*	1.00[0.98-1.02];0.8231	1.01[1.00-1.02];0.1025	0.97[0.92-1.03];0.3646
Platelet-to-lymphocyte ratio	1.000[1.000-1.000];0.0261*	1.000[1.000-1.001];0.5907	1.000[1.000-1.000];0.1087	1.000[0.999-1.001];0.9257
Aspartate transaminase-to-alanine transaminase ratio	1.03[1.01-1.04];<0.0001***	1.01[0.97-1.06];0.6492	1.09[1.04-1.14];0.0004***	0.96[0.71-1.29];0.7683
Triglyceride glucose index	0.97[0.83-1.13];0.6770	0.59[0.41-0.85];0.0040**	0.85[0.66-1.09];0.2061	0.55[0.30-1.02];0.0594
Urea-to-creatinine ratio	1.000[0.999-1.001];0.9657	1.000[0.997-1.002];0.9081	1.00[1.00-1.01];0.2003	1.01[1.00-1.01];0.0007***
Monocyte-to-lymphocyte ratio	1.07[0.99-1.16];0.0998	0.99[0.79-1.23];0.9303	1.20[1.02-1.43];0.0305*	1.07[0.69-1.67];0.7559
***Complete blood counts***				
Hb, g/dL	0.97[0.95-1.00];0.0539	1.00[0.94-1.06];0.9969	0.95[0.91-1.00];0.0543	1.19[1.05-1.34];0.0072**
SD of Hb	1.58[1.44-1.74];<0.0001***	1.51[1.23-1.85];0.0001***	1.52[1.29-1.79];<0.0001***	1.94[1.36-2.76];0.0003***
Mean corpuscular volume, fL	1.00[0.99-1.01];0.9942	1.01[1.00-1.03];0.1496	1.00[0.98-1.01];0.5845	1.03[1.00-1.07];0.0470*
Eosinophil, x10^9/L	0.74[0.58-0.93];0.0105*	0.84[0.54-1.31];0.4333	0.81[0.53-1.23];0.3143	1.14[0.49-2.64];0.7632
Lymphocyte, x10^9/L	0.98[0.92-1.05];0.6221	1.08[0.97-1.20];0.1514	0.96[0.83-1.10];0.5296	1.07[0.78-1.47];0.6699
Metamyelocyte, x10^9/L	0.87[0.70-1.07];0.1830	0.78[0.46-1.31];0.3392	0.71[0.40-1.28];0.2549	0.74[0.21-2.63];0.6383
Monocyte, x10^9/L	1.24[1.06-1.45];0.0078**	1.23[0.87-1.73];0.2362	1.51[1.14-2.00];0.0045**	1.65[0.88-3.11];0.1194
Neutrophil, x10^9/L	1.02[1.01-1.04];0.0013**	1.01[0.98-1.05];0.4025	1.03[1.01-1.06];0.0129*	1.02[0.96-1.08];0.5817
White blood count, x10^9/L	1.02[1.01-1.03];<0.0001***	1.02[1.00-1.05];0.1069	1.03[1.00-1.05];0.0187*	1.02[0.97-1.08];0.4938
Mean cell haemoglobin, pg	0.99[0.97-1.01];0.2267	1.01[0.97-1.05];0.6190	0.96[0.94-0.99];0.0133*	1.05[0.97-1.14];0.1981
Myelocyte, x10^9/L	0.93[0.84-1.02];0.1131	0.88[0.66-1.18];0.3893	0.83[0.66-1.04];0.1098	0.83[0.50-1.39];0.4823
Platelet, x10^9/L	1.001[1.000-1.001];0.0388*	1.001[1.000-1.002];0.2819	1.002[1.001-1.003];0.0015**	1.002[0.999-1.004];0.1452
Red blood count, x10^12/L	0.93[0.86-1.01];0.0818	0.93[0.78-1.10];0.3688	0.91[0.79-1.06];0.2409	1.26[0.89-1.78];0.1925
Hematocrit, L/L	0.30[0.11-0.80];0.0163*	0.75[0.09-6.67];0.7997	0.18[0.03-1.13];0.0671	566.25[4.88-65751.46];0.0090**
***Renal and liver functions***				
Potassium, mmol/L	0.91[0.80-1.03];0.1211	1.03[0.79-1.35];0.8120	0.87[0.69-1.09];0.2157	0.92[0.56-1.52];0.7440
Urate, mmol/L	1.54[0.72-3.30];0.2671	5.04[0.95-26.62];0.0570	1.71[0.32-9.18];0.5314	0.65[0.02-19.52];0.8040
Albumin, g/L	0.97[0.96-0.98];<0.0001***	0.98[0.96-1.00];0.0540	0.98[0.96-0.99];0.0061**	1.04[0.99-1.09];0.1011
Sodium, mmol/L	0.97[0.96-0.98];<0.0001***	0.97[0.95-1.00];0.0227*	0.95[0.92-0.97];0.0001***	0.97[0.91-1.05];0.4786
Urea, mmol/L	0.99[0.97-1.02];0.5540	1.02[0.98-1.06];0.4164	0.98[0.93-1.03];0.4672	1.00[0.89-1.13];0.9939
Protein, g/L	0.99[0.99-1.00];0.0001***	1.00[0.99-1.01];0.8901	0.99[0.99-1.00];0.0087**	1.00[0.98-1.02];0.9227
Bilirubin, umol/L	1.003[1.002-1.005];<0.0001***	1.00[1.00-1.01];0.5730	1.00[1.00-1.01];0.9330	1.00[0.99-1.01];0.9730
Creatinine, umol/L	1.001[1.000-1.001];0.2210	1.000[0.998-1.002];0.7707	1.001[0.999-1.002];0.2969	1.00[0.99-1.00];0.4195
SD of creatinine	1.001[1.000-1.001];0.0001***	1.001[1.000-1.002];0.0719	1.001[1.000-1.001];0.0974	1.000[0.998-1.002];0.7760
Aspartate transaminase, U/L	1.001[1.001-1.001];<0.0001***	1.000[0.998-1.002];0.8846	1.001[0.999-1.002];0.4383	0.99[0.98-1.01];0.2798
SD of aspartate transaminase	1.001[1.000-1.001];<0.0001***	1.000[1.000-1.001];0.2117	1.001[1.000-1.002];0.0155*	1.001[0.997-1.004];0.6500
Alkaline phosphatase, U/L	1.001[1.001-1.002];<0.0001***	1.000[0.999-1.002];0.7097	1.000[0.999-1.001];0.4551	1.00[0.99-1.00];0.2454
SD of alkaline phosphatase	1.002[1.002-1.003];<0.0001***	1.002[1.001-1.003];0.0014**	1.003[1.002-1.003];<0.0001***	1.002[1.000-1.004];0.0359*
Alanine transaminase, U/L	1.000[1.000-1.001];0.3737	1.000[0.998-1.002];0.8684	0.999[0.995-1.002];0.3897	0.99[0.98-1.00];0.0914
SD of alanine transaminase	1.001[1.001-1.002];<0.0001***	1.001[1.000-1.002];0.0177*	1.001[1.000-1.003];0.0741	1.00[0.99-1.00];0.5347
***Lipid, iron, and calcium profile***				
Total iron-binding capacity, L	1.00[0.98-1.02];0.9971	1.00[0.95-1.05];0.9678	0.99[0.95-1.04];0.7518	3.87[0.00-Inf];0.9993
VitaminB12, pmol/L	0.999[0.998-1.000];0.1739	1.00[0.99-1.00];0.1813	1.000[0.998-1.002];0.7853	1.00[0.99-1.01];0.8806
Folate, ng/mL	1.00[0.97-1.03];0.9877	1.04[0.97-1.12];0.2752	1.00[0.96-1.05];0.8835	1.04[0.95-1.14];0.3791
Ferritin, pmol/L	1.000[1.000-1.000];0.4438	1.000[1.000-1.000];0.3647	1.000[1.000-1.000];0.2525	1.000[1.000-1.001];0.1003
Calcium, mmol/L	0.64[0.39-1.06];0.0826	0.41[0.13-1.26];0.1212	0.91[0.36-2.28];0.8345	1.40[0.20-9.84];0.7350
SD of calcium	12.70[5.21-30.97];<0.0001***	2.10[0.13-34.34];0.6015	4.69[1.00-22.02];0.0499*	2.32[0.04-132.03];0.6840
Phosphate, mmol/L	0.83[0.59-1.18];0.3059	0.79[0.36-1.74];0.5548	0.98[0.50-1.90];0.9464	0.61[0.14-2.69];0.5115
SD of phosphate	5.97[3.23-11.04];<0.0001***	7.44[1.81-30.57];0.0054**	3.91[0.74-20.76];0.1098	18.22[0.41-815.46];0.1345
***Glycemic and clotting profile***				
Triglyceride, mmol/L	0.96[0.88-1.05];0.3264	0.74[0.56-0.99];0.0436*	0.90[0.78-1.04];0.1556	0.61[0.35-1.09];0.0933
SD of triglyceride	0.84[0.64-1.11];0.2196	0.20[0.04-1.02];0.0526	0.69[0.41-1.17];0.1655	0.54[0.08-3.53];0.5213
Glucose, mmol/L	1.02[0.99-1.04];0.1738	1.00[0.94-1.05];0.9179	0.99[0.95-1.03];0.5861	0.90[0.79-1.04];0.1558
SD of glucose	1.06[1.02-1.11];0.0032**	1.07[0.98-1.16];0.1337	1.07[1.00-1.14];0.0580	1.07[0.92-1.23];0.3816
High sensitive troponin-I, ng/L	1.000[1.000-1.000];0.9239	1.000[1.000-1.000];0.1181	1.000[1.000-1.000];0.9382	1.000[1.000-1.001];0.1785
SD of high sensitive troponin-I	1.000[1.000-1.000];0.7182	1.000[1.000-1.000];0.0044**	1.000[0.999-1.001];0.9978	1.000[1.000-1.001];0.2563
APTT, second	1.01[1.00-1.03];0.1426	0.98[0.93-1.03];0.3733	0.99[0.95-1.03];0.6325	0.98[0.89-1.08];0.6750
SD of APTT	1.05[1.02-1.08];0.0006***	0.99[0.90-1.10];0.8880	1.01[0.90-1.13];0.8927	1.10[0.86-1.42];0.4432
Lactate dehydrogenase, U/L	1.000[1.000-1.000];<0.0001***	1.000[1.000-1.001];0.0165*	1.000[1.000-1.001];0.0001***	1.000[0.999-1.001];0.6208
SD of lactate dehydrogenase	1.000[1.000-1.000];<0.0001***	1.000[1.000-1.001];0.0229*	1.002[1.001-1.002];<0.0001***	1.002[1.000-1.003];0.0056**
Total cholesterol, mmol/L	0.98[0.90-1.07];0.6769	0.92[0.75-1.13];0.4335	1.05[0.90-1.22];0.5598	1.42[1.00-2.00];0.0490*
SD of total cholesterol	1.14[0.84-1.55];0.3905	0.93[0.42-2.07];0.8677	1.23[0.73-2.07];0.4394	2.73[0.81-9.19];0.1058
Low-density lipoprotein, mmol/L	1.00[0.90-1.11];0.9905	0.96[0.76-1.22];0.7514	1.14[0.96-1.37];0.1398	1.67[1.14-2.45];0.0091**
SD of low-density lipoprotein	1.22[0.84-1.78];0.2952	1.13[0.45-2.83];0.7919	1.66[0.77-3.55];0.1928	7.64[1.84-31.77];0.0051**
High-density lipoprotein, mmol/L	0.93[0.73-1.19];0.5774	1.27[0.77-2.11];0.3530	0.97[0.62-1.50];0.8772	1.96[0.82-4.71];0.1309
SD of high-density lipoprotein	2.42[0.85-6.87];0.0971	1.55[0.11-21.84];0.7461	1.32[0.19-9.12];0.7758	1.09[0.01-164.28];0.9719

* for p≤ 0.05, ** for p ≤ 0.01, *** for p ≤ 0.001; HR: Hazard ratio; CI: Confidence interval; APTT: applied partial thromboplastin test; PD-1: Programmed death 1 inhibitors; PD-L1: programmed death 1 ligand inhibitors

Univariable Cox regression identified significant predictors of the primary composite outcome and all-cause mortality before and after propensity score matching (Table 3). Compared with PD-1 inhibitor treatment, PD-L1 inhibitor treatment was significantly associated with a lower risk of composite outcome both before (hazard ratio [HR]: 0.32, 95% CI: [0.18-0.59], P value=0.0002***) and after matching (HR: 0.34, 95% CI: [0.18-0.65], P value=0.001**), and lower all-cause mortality risk before matching (HR: 0.77, 95% CI: [0.64-0.93], P value=0.0078**) and after matching (HR: 0.80, 95% CI: [0.65-1.00], P value=0.0463).

More PD-L1 expenditure (HR:1.000; 95% CI: 1.000-1.000; P value=0.0528), shorter PD-L1 inhibitors duration (HR:0.99; 95% CI: 0.99-1.00; P value=0.0263*), more PD-1 expenditure, HKD (HR:1.000; 95% CI: 1.000-1.001; P value=0.0018**), and shorter PD-1 inhibitors duration (HR:0.998; 95% CI: 0.997-0.999; P value=0.0007***) were associated with new onset cardiac composite outcome in the matched cohort. Significant laboratory examinations significantly associated with new onset cardiac composite outcome include higher levels of mean corpuscular volume (HR:1.03; 95% CI: 1.00-1.07; P value=0.0470*), hematocrit (HR:566.25; 95% CI: 4.88-65751.46; P value=0.0090**), HbA1c (HR:1.19; 95% CI: 1.05-1.34; P value=0.0072**), total cholesterol (HR:1.42; 95% CI: 1.00-2.00; P value=0.0490*), and low-density lipoprotein (HR:1.67; 95% CI: 1.14-2.45; P value=0.0091**).

**Fig. 4:**
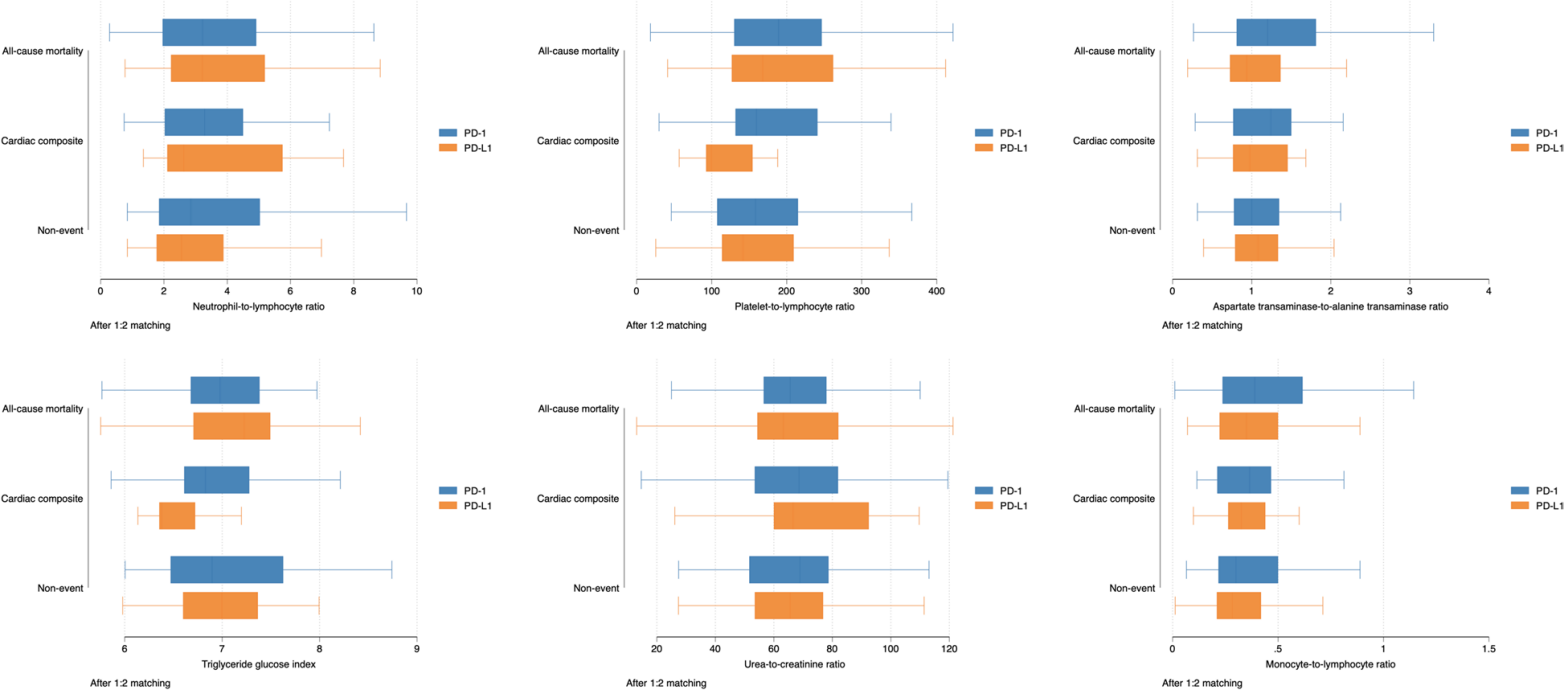
Boxplots of significant laboratory examination variability stratified by PD-1/PD-L1 inhibitor use and development of the composite outcome in the matched cohort

In addition, greater variability in laboratory tests, including the standard deviations (SD) of alkaline phosphatase (HR:1.002; 95% CI: 1.000-1.004; P value=0.0359*), HbA1c (HR:1.94; 95% CI: 1.36-2.76; P value=0.0003***), lactate dehydrogenase (HR:1.002; 95% CI: 1.000-1.003; P value=0.0056**), and low-density lipoprotein (HR:7.64; 95% CI: 1.84-31.77; P value=0.0051**) were significantly associated with the composite outcome. The boxplots of significant measures of variability stratified by PD-1/PD-L1 inhibitor treatment in the matched cohort are shown in Fig. 4.

In addition, multivariable Cox regression models (Table 4) with multiple adjustments with significant demographics, past comorbidities, and non-PD-L1/PD-1 drugs confirmed the protection effects of PD-L1 than PD-1 for adverse study outcomes (HR<1, P < 0.05).

**Table 4:** Comparisons of hospitalization characteristics before and after PD1/PD-L1 treatment in patients with new onset heart failure, acute myocardial infarction, atrial fibrillation, and atrial flutter

Hospitalization characteristics	Before matching			After 1:1 matching		
	Before treatment Mean(SD);N or Count(%)	After treatment Mean(SD);N or Count(%)	P value	Before treatment Mean(SD);N or Count(%)	After treatment Mean(SD);N or Count(%)	P value
***Heart failure (N = 244)***				***Heart failure*** (N = 75)		
Average readmission interval between episodes, days	299.3(680.4);n = 202	45.5(95.9);n = 244	<0.0001***	358.6(922.4);n = 61	38.1(45.2);n = 75	<0.0001***
No. of episodes	8.8(7.4);n = 202	13.8(12.3);n = 244	<0.0001***	11.0(7.8);n = 61	12.9(9.5);n = 75	<0.0001***
Hospital stay, days	26.6(35.0);n = 202	40.6(36.0);n = 244	<0.0001***	33.7(38.8);n = 61	39.9(39.6);n = 75	<0.0001***
***Acute myocardial infarction (N = 38)***				***Acute myocardial infarction*** (N = 8)		
Average readmission interval between episodes, days	498.3(1094.7);n = 32	42.1(53.5);n = 38	<0.0001***	1576.1(2728.8);n = 6	38.9(16.4);n = 8	<0.0001***
No. of episodes	6.4(5.7);n = 32	13.2(8.9);n = 38	<0.0001***	5.8(3.9);n = 6	17.4(11.6);n = 8	<0.0001***
Hospital stay, days	24.5(28.9);n = 32	34.3(30.4);n = 38	<0.0001***	11.5(6.4);n = 6	20.8(12.3);n = 8	<0.0001***
***Atrial fibrillation (N = 54)***				***Atrial fibrillation*** (N = 8)		
Average readmission interval between episodes, days	383.9(905.9);n = 46	43.4(48.0);n = 54	<0.0001***	715.5(1861.5);n = 6	49.4(45.0);n = 8	<0.0001***
No. of episodes	9.2(8.8);n = 46	12.5(10.2);n = 54	<0.0001***	15.6(11.1);n = 6	9.0(5.0);n = 8	<0.0001***
Hospital stay, days	28.7(29.3);n = 46	43.6(54.6);n = 54	<0.0001***	30.4(27.5);n = 6	23.4(25.5);n = 8	<0.0001***
***Atrial flutter (N = 6)***				***Atrial flutter (N = 2)***		
Average readmission interval between episodes, days	190.2(175.8);n = 5	42.5(47.1);n = 6	<0.0001***	-	-	-
No. of episodes	3.8(2.2);n = 5	11.5(7.8);n = 6	<0.0001***	-	-	-
Hospital stay, days	28.0(32.5);n = 5	33.3(22.1);n = 6	<0.0001***	-	-	-

* for p≤ 0.05, ** for p ≤ 0.01, *** for p ≤ 0.001; PD-1: Programmed death 1 inhibitors; PD-L1: programmed death 1 ligand inhibitors

### Healthcare utilization before and after treatment with PD-1/PD-L1 inhibitors

Longer overall cumulative hospital stay (HR:1.01; 95% CI: 1.00-1.01; P value=0.0040**) and longer hospital stay after PD-1/PD-L1 drug use (HR:1.01; 95% CI: 1.00-1.01; P value=0.0040**) were significantly associated with the composite outcome. Furthermore, hospitalization characteristics before and after PD-1/PD-L1 treatment were compared in the subset of patients who developed the adverse outcomes, in both the unmatched and matched cohorts (Table 4). Patients who developed cardiovascular complications had a shorter average readmission interval, a higher number of hospitalizations and a longer duration of hospital stay after PD-1/PD-L1 treatment (P < 0.0001).

### Sensitivity analysis

Supplementary Table 7 presented the adjusted hazard ratios (and 95% CIs) of PD-L1 vs. PD-1 with cause-specific and subdistribution hazard competing risk analysis models for new onset cardiac composite and mortality outcomes after 1:2 propensity score matching. Supplementary Table 8 presented the hazard ratios for associations of PD-L1 vs. PD-1 using Cox proportional hazard model for adverse new onset cardiac composite and mortality outcome in the 1:2 matched cohort, with half-year lag time. Supplementary Table 9 presented the risk of incident new onset cardiac composite and mortality outcomes associated with treatment of PD-L1 vs. PD-1 with multiple matching adjustment approaches including propensity score stratification, high-dimensional propensity score matching, and propensity score matching with inverse probability of treatment weighting. The above analysis confirmed the protective effects of PD-L1 treatment over PD-1 treatment on new onset cardiac complications and mortality risks (HR<1, P value<0.05).

### Prediction strength of subclinical inflammatory biomarkers

**Fig. 5:**
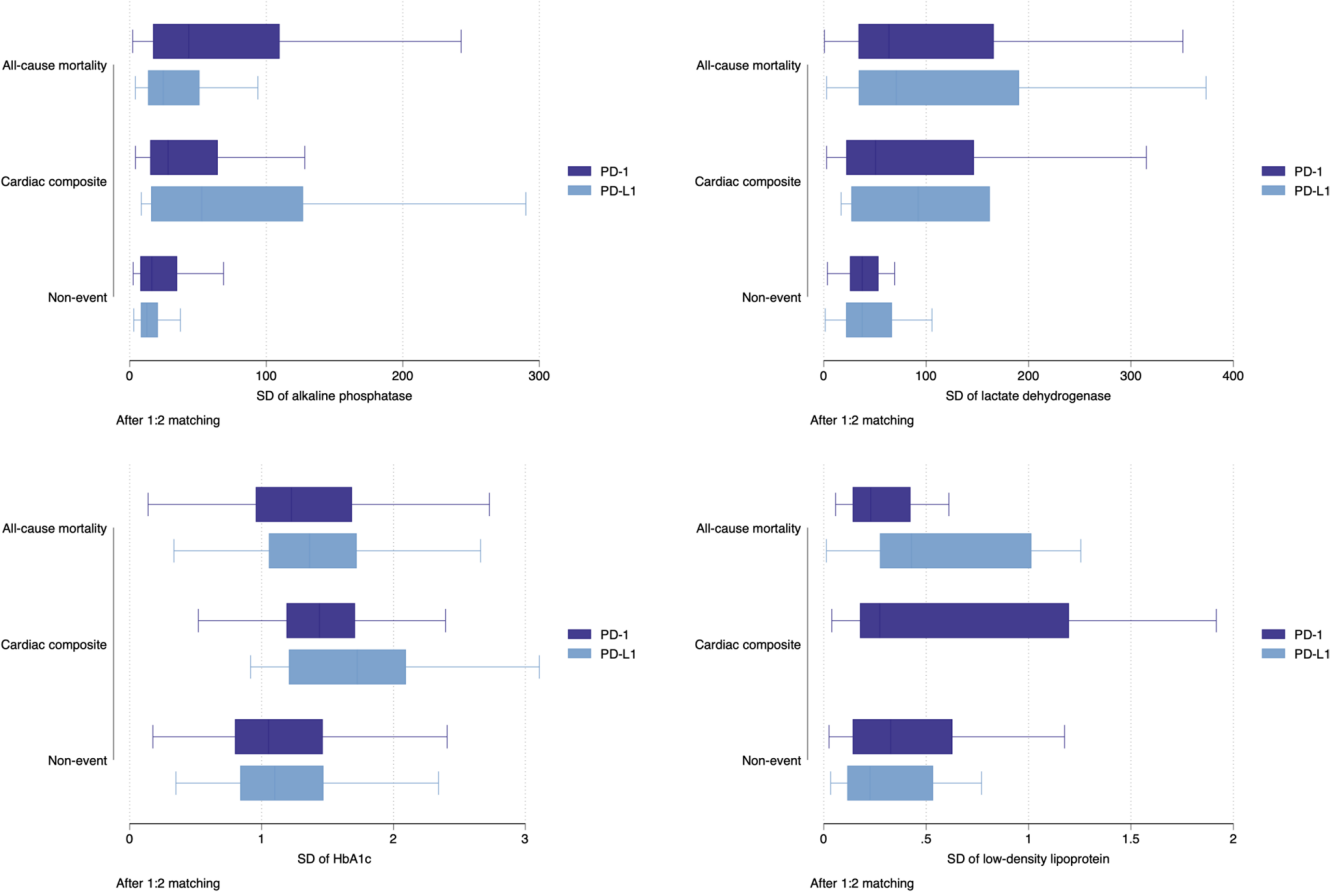
Boxplots of subclinical inflammatory biomarkers stratified by PD-1/PD-L1 inhibitor use and development of the composite outcome in the matched cohort

In the matched cohort, higher aspartate transaminase-to-alanine transaminase ratio (HR: 1.09, 95% CI: [1.04-1.14], P value=0.0004) and higher monocyte-to-lymphocyte ratio (HR: 1.2, 95% CI: [1.02-1.43], P value=0.0305) were significantly associated with all-cause mortality. A higher urea-to-creatinine ratio (HR: 1.01, 95% CI: [1.00-1.01], P value=0.0007) was significantly associated with new onset cardiac composite outcome (Table 5). The boxplots of inflammatory biomarkers stratified by PD-1/PD-L1 inhibitor use and development of adverse outcomes are shown in Fig. 5.

**Table 5:** Multivariable univariable Cox regression models for new onset cardiac complication outcome and all-cause mortality in the matched cohort

**Model 1**	**All-cause mortality** **HR [95% CI];P value**	**Composite outcome** **HR [95% CI];P value**
**Characteristics**		
PD-L1 v.s. PD-1	0.80[0.64-0.99];0.0389*	0.34[0.18-0.64];0.0009***
PD-L1 expenditure, HKD	1.000[1.000-1.0001];<0.0001***	1.000[1.000-1.000];0.0592
Total PD-L1 dose amount, mg	1.000[1.000-1.000];0.0728	1.000[1.000-1.000];0.2615
PD-L1 inhibitors duration, days	1.00[0.99-1.001];<0.0001***	0.99[0.99-1.00];0.0301*
PD-1 expenditure, HKD	1.000[1.000-1.0001];<0.0001***	1.000[1.000-1.0001];0.0014**
Total PD-1 dose amount (mg)	1.000[1.000-1.0001];0.0005***	1.000[1.000-1.000];0.1100
PD-1 inhibitors duration, days	0.998[0.997-0.998];<0.0001***	0.998[0.997-0.999];0.0006***
**Model 2**	**All-cause mortality** **HR [95% CI];P value**	**Composite outcome** **HR [95% CI];P value**
**Characteristics**		
PD-L1 v.s. PD-1	0.79[0.61-0.99];0.0366*	0.34[0.16-0.65];0.0009***
PD-L1 expenditure, HKD	1.000[1.000-1.0001];<0.0001***	1.000[1.000-1.000];0.0528
Total PD-L1 dose amount, mg	1.000[1.000-1.000];0.0740	1.000[1.000-1.000];0.2448
PD-L1 inhibitors duration, days	1.00[0.99-1.00];<0.0001***	0.99[0.99-1.00];0.0295*
PD-1 expenditure, HKD	1.000[1.000-1.0001];<0.0001***	1.000[1.000-1.0001];0.0012**
Total PD-1 dose amount (mg)	1.000[1.000-1.0001];0.0007***	1.000[1.000-1.000];0.1334
PD-1 inhibitors duration, days	0.998[0.997-0.998];<0.0001***	0.998[0.997-0.999];0.0007***
**Model 3**	**All-cause mortality** **HR [95% CI];P value**	**Composite outcome** **HR [95% CI];P value**
**Characteristics**		
PD-L1 v.s. PD-1	0.81[0.64-0.98];0.0322*	0.33[0.18-0.63];0.0007***
PD-L1 expenditure, HKD	1.000[1.000-1.0001];<0.0001***	1.000[1.000-1.0001];0.0433*
Total PD-L1 dose amount, mg	1.000[1.000-1.000];0.0733	1.000[1.000-1.000];0.2482
PD-L1 inhibitors duration, days	1.00[1.00-1.001];<0.0001***	0.99[0.99-1.00];0.0282*
PD-1 expenditure, HKD	1.000[1.000-1.0001];<0.0001***	1.000[1.000-1.0001];0.0020**
Total PD-1 dose amount (mg)	1.000[1.000-1.0001];0.0008***	1.000[1.000-1.000];0.1673
PD-1 inhibitors duration, days	0.998[0.997-0.998];<0.0001***	0.998[0.997-0.999];0.0008***

Model 1 adjusted for significant demographics

Model 2 adjusted for significant demographics, and past comorbidities

Model 3 adjusted for significant demographics, past comorbidities, and non-PD-L1/PD-1 medications

* for p≤ 0.05, ** for p ≤ 0.01, *** for p ≤ 0.001; HR: Hazard ratio; CI: Confidence interval; APTT: applied partial thromboplastin test; PD-1: Programmed death 1 inhibitors; PD-L1: programmed death 1 ligand inhibitors; IR: incidence rate

## Discussion

The main findings are that: (i) the incidence of cardiovascular complications after PD-1 or PD-L1 inhibitor use was 16% in this territory-wide cohort of Chinese patients from Hong Kong, (ii) multivariable Cox regression showed older age, a shorter average readmission interval and a higher number of hospital admissions were significant predictors of cardiovascular complications and (iii) patients who developed cardiovascular complications had shorter average readmission interval and higher number of hospitalizations after treatment with PD-1/PD-L1 inhibitors.

Cardiac involvement in PD1 or PD-L1 inhibitors is variable, and can potentially affect the conduction system, myocardium or pericardium [17]. Thus, heart block [18], Takotsubo cardiomyopathy [19], myocarditis [20, 21] and pericarditis [22] have been reported. A meta-analysis performed in 2018 found that anti-PD-1/PD-L1-related fatalities were often from pneumonitis (333 [35%]), hepatitis (115 [22%]), and neurotoxic effects (50 [15%]). Combination PD-1/ cytotoxic T-lymphocyte-associated protein 4 (CTLA-4) deaths were frequently from colitis (32 [37%]) and myocarditis (22 [25%]) [23]. In an analysis of the World Health Organization global database of adverse drug reactions in 2019, 2.1% of 106,025 patients receiving PD-1 or PD-L1 inhibitors had cardiovascular complications [9]. However, previous studies have largely been limited to case reports [18, 24], case series [25], single-center studies [26] or small registries [27, 28]. In this territory-wide study from Hong Kong, we found that cardiovascular complications occurred in 16% of all patients receiving PD1 or PD-L1 inhibitors. Of these, the commonest is heart failure. Previously, acute heart failure has been described in the context of myocarditis [29], but heart failure without myocarditis has also been reported [30]. Our study also identified cases of acute myocardial infarction following the initiation of PD-1/PD-L1 inhibitor therapy. Such findings would be in keeping with coronary toxicity that has been reported in the context of PD-1 inhibitor therapy [27].

It is worth noting that the present study is the first to compare the cardiotoxicity between PD-1 and PD-L1 inhibitor treatment. The reason for the protective effect of PD-L1 inhibitor to be stronger than PD-1 inhibitor may be the stronger immune-mediated effects of PD-1 inhibitor. Contrary to PD-1 inhibitor, which blocks the interaction between PD-1 and both PD-L1 and PD-L2, PD-L1 inhibitor only blocks the interaction between PD-1 and PD-L1. [31]Although cancer may escape from immune-mediated detection via the PD-1/ PD-L2 axis under PD-L1 inhibitor treatment, it also implies that PD-L1 inhibitor may have weaker autoimmunity, thus resulting in less immune-mediated cardiotoxicity. [32] Furthermore, patients who developed cardiovascular complications had a shorter average readmission interval and more hospitalizations, which is due to the deterioration in patients’ general health and treatment needed for the complications. For example, acute exacerbation of heart failure is known to be common reasons for frequent admissions. The presence of adverse cardiovascular events is detrimental to the overall health of cancer patients who are already frail, which may lead to the decline in health and related frequent admissions, which demonstrates the critical effects of cardiovascular complications on patients’ physical health and quality of life.

Interestingly, our study did not identify any patients with myocarditis after treatment with PD-1 or PD-L1 inhibitors. Moreover, within the excluded patients with prior cardiovascular complications, none developed subsequent myocarditis. In 964 patients attending Massachusetts General Hospital, the incidence of myocarditis was 1.1% (n = 35) [28]. In this cohort, myocarditis was more frequently observed in patients with pre-existing cardiovascular comorbidities. Nevertheless, another study using the VigiBase database found 101 cases of severe myocarditis, of which 75% of the myocarditis cases did not have pre-existing cardiovascular disease [33]. A single-center study of 283 patients from China found only 3 cases (1.1%) of myocarditis, with variable presentations such as palpitations, dyspnea, and fatigue, or asymptomatic with incidental finding of grade 3 atrioventricular block and premature ventricular complexes on the electrocardiogram [26]. In a pooled, retrospective review of three trials including 448 patients with advanced melanoma receiving PD-1/PD-L1 inhibitor therapy, no cases of myocarditis were identified [34]. In association with myocarditis, different investigators have reported the presence of conduction abnormalities in the form of atrioventricular block [18, 25, 26, 35].

### Limitations

There are some limitations of this study that should be acknowledged. Firstly, this was an administrative database study, and therefore cancer staging details could not be extracted. Secondly, under-coding or miscoding remains a possibility as with studies of a similar nature. Thirdly, although no cases of myocarditis were found. As our study relies on ICD-9 coding, this might be due to under-coding. Alternatively, missed cases by the clinicians and subclinical myocarditis leading to heart failure are possible. Therefore, further studies accounting for parameters that may uncover undiagnosed myocarditis, such as creatine kinase, is needed to ensure that the diagnosis of myocarditis is not missed.

## Conclusions

Compared with PD-1 inhibitor use, PD-L1 inhibitor use was significantly associated with lower risks of cardiac complications and all-cause mortality both before and after propensity score matching. Patients who developed cardiovascular complications had shorter average readmission intervals and a higher number of hospitalizations after treatment with PD-1/PD-L1 inhibitors.

## Supplementary Information

**Additional file 1.**

## Data Availability

Data availability upon request to the corresponding author.

## Declarations

The need for informed consent was waived by the Ethics Committee owing to the retrospective and observational nature of the study.
