# Supplementary material for: Adverse Cardiovascular Complications following prescription of programmed cell death 1 (PD-1) and programmed cell death ligand 1 (PD-L1) inhibitors: a propensity-score matched Cohort Study with competing risk analysis
Source: Cardiooncology. 2022 Mar 17;8:5. doi: 10.1186/s40959-021-00128-5 (PMC8928662; doi:10.1186/s40959-021-00128-5)
Supplement: Supplementary file 1 — Additional file 1. [file 40959_2021_128_MOESM1_ESM.pdf]

Supplementary Table 1. PD-1 and PD-L1 inhibitor drugs.

PD-1 inhibitors	PD-L1 inhibitors
Pembrolizumab	Atezolizumab (Tecentriq)
Nivolumab	Avelumab (Bavencio)
Cemiplimab	Durvalumab (Imfinzi)
Spartalizumab	KN035
Camrelizumab	CK-301
Sintilimab (IBI308)	AUNP12
Tislelizumab (BGB-A317)	CA-170
Toripalimab (JS 001)	BMS-986189
Dostarlimab (TSR-042, WBP-285)	
INCMGA00012 (MGA012)	
AMP-224	
AMP-514 (MEDI0680)	

Supplementary Table 2. Codes for comorbidities.

Diabetes mellitus	250 250.01 250.02 250.03 250.1 250.11 250.12 250.13 250.2 250.21 250.22 250.23 250.3 250.31 250.32 250.33 250.4 250.41 250.42 250.43 250.5 250.51 250.52 250.53 250.6 250.61 250.62 250.63 250.7 250.71 250.72 250.73 250.8 250.81 250.82 250.83 250.9 250.91 250.92 250.93
Hypertension	401 401.1 401.9 402 402.01 402.1 402.11 402.9 402.91 403 403.01 403.1 403.11 403.9 403.91 404 404.01 404.02 404.03 404.1 404.11 404.12 404.13 404.9 404.91 404.92 404.93 405 405.01 405.09 405.1 405.11 405.19 405.9 405.91 405.99 437.2
Heart failure	428 428 428.1 428.2 428.2 428.21 428.22 428.23 428.3 428.3 428.31 428.32 428.33 428.4 428.4 428.41 428.42 428.43

428.9 398.91 402.01 402.11 402.91 404.01 404.03 404.11 404.13 404.91 404.93
Atrial fibrillation 427.31 429.4
Maligt dysrhythmia 426 426.12 426.13 426.51 426.52 426.54 427.1 427.4 427.41 427.42 427.5
Atrial flutter 427.32
Liver diseases 456 456.1 456.2 572.2 572.3 572.4 572.8 571.4 571.5 571.6
Chronic obstructive pulmonary disease 490 491 492 493 494 495 496 491.1 491.2 491.21 491.22 491.8 491.9 492.8 493.01 493.02 493.1 493.11 493.12 493.2 493.21 493.22 493.8 493.81 493.82 493.9 493.91 493.92 494.1 495.1 495.2 495.3 495.4 495.5 495.6 495.7 495.8 495.9
Gastrointestinal bleeding 531 531.2 531.4 531.6 532 532.2 532.4 532.6 533 533.2 533.4 533.6 534 534.2 534.4 534.6 535.01 535.11 535.21 535.31 535.41 535.51 535.61 535.71 562.02 562.03 562.12 562.13 569.3 569.85 569.86 578 578.1 578.9
Hip fractures/accident falls 805 805 805 805.01 805.02 805.03 805.04 805.05 805.06 805.07 805.08 805.1 805.1 805.11 805.12 805.13 805.14 805.15 805.16 805.17 805.18 805.2 805.3 805.4 805.5 805.6 805.7 805.8 805.9 812 812 812 812.01 812.02 812.03 812.09 812.1 812.1 812.11 812.12 812.13 812.19 812.2 812.2 812.21 812.3 812.3 812.31 812.4 812.4 812.41 812.42 812.43 812.44 812.49 812.5 812.5 812.51 812.52 812.53 812.54 812.59 813 813 813 813.01 813.02 813.03 813.04 813.05 813.06 813.07 813.08 813.1 813.1 813.11 813.12 813.13 813.14 813.15 813.16 813.17 813.18 813.2 813.2 813.21 813.22 813.23 813.3 813.3 813.31 813.32 813.33 813.4 813.4 813.41 813.42 813.43 813.44 813.45 813.46 813.47 813.5 813.5 813.51 813.52 813.53 813.54 813.8 813.8 813.81 813.82 813.83 813.9 813.9 813.91 813.92 813.93 814 814 814 814.01 814.02 814.03 814.04 814.05 814.06 814.07 814.08 814.09 814.1 814.1 814.11 814.12 814.13 814.14 814.15 814.16 814.17 814.18 814.19 820 820 820 820.01 820.02 820.03 820.09 820.1 820.1 820.11 820.12 820.13 820.19 820.2 820.2 820.21 820.22 820.3 820.3 820.31 820.32 820.8 820.9 170 170 170.1 170.2 170.3 170.4 170.5 170.6 170.7 170.8 170.9 710 710.1 710.4 714 714.1 714.2 714.81 725 E880

E880.0 E880.1 E880.9 E881 E881.0 E881.1 E882 E883 E883.0 E883.1 E883.2 E883.9 E884 E884.0 E884.1 E884.2 E884.3 E884.4 E884.5 E884.6 E884.9 E885 E885.0 E885.1 E885.2 E885.3 E885.4 E885.9 E886 E886.0 E886.9 E887 E888 E888.0 E888.1 E888.8 E888.9
Malignant dysrhythmia 426 426.12 426.13 426.51 426.52 426.54 427.1 427.4 427.41 427.42 427.5
Renal diseases 582 582 582.1 582.2 582.4 582.8 582.81 582.89 582.9 583 583 583.1 583.2 583.4 583.6 583.7 585 585.1 585.2 585.3 585.4 585.5 585.6 585.9 586 588 588 588.1 588.8 588.81 588.89 588.9
Endocrine diseases 259 259 259.1 259.2 259.3 259.4 259.5 259.5 259.51 259.52 259.8 259.9
Peripheral vascular disease 250.7 443.9 443 443.1 443.2 443.21 443.22 443.23 443.24 443.29 443.8 443.81 443.82 443.89 441 443.9 785.4 V43.4
Stroke/transient ischemic attack 435 435.1 435.2 435.3 435.8 435.9 433.81 433.91 434 436 437 437.1 433.31 433.01 434.01 434.1 434.11 434.9 434.91 437.2 437.3 437.4 437.5 437.6 437.7 437.8 437.9
Gastrointestinal bleeding 531 531.2 531.4 531.6 532 532.2 532.4 532.6 533 533.2 533.4 533.6 534 534.2 534.4 534.6 535.01 535.11 535.21 535.31 535.41 535.51 535.61 535.71 562.02 562.03 562.12 562.13 569.3 569.85 569.86 578 578.1 578.9
Ischemic heart disease 410.01 410.02 410.1 410.11 410.12 410.2 410.21 410.22 410.3 410.31 410.32 410.4 410.41 410.42 410.5 410.51 410.52 410.6 410.61 410.62 410.7 410.71 410.72 410.8 410.81 410.82 410.9 410.91 410.92 411 411.1 411.8 411.81 411.89 413 413.1 413.9 414 414.01 414.02 414.03 414.04 414.05 414.06 414.07 414.1 414.11 414.12 414.19 414.2 414.3 414.4 414.8 414.9 410 412
Cancer 140-239

Supplementary Table 3. Logistic regression analysis of confounding characteristics before conducting propensity score matching (Mortality as outcome).

Variables	Coef.	St.Err.	t-value	p-value	[95% Conf	Interval]	Sig
PD-L1 v.s. PD-1	.45	.067	-5.35	0	.336	.603	***
Male gender	.887	.096	-1.11	.269	.716	1.097	
Baseline age, years	.998	.004	-0.46	.645	.99	1.006	
Charlson standard comorbidity index	1.11	.02	5.82	0	1.072	1.15	***
Hypertension	1.1	.188	0.56	.577	.787	1.537	
Liver diseases	1.404	.253	1.88	.06	.986	1.998	*
Hip fractures/accident falls	1.335	.369	1.04	.297	.776	2.295	
Renal diseases	.887	.127	-0.84	.401	.671	1.173	
Diabetes mellitus	.91	.19	-0.45	.651	.604	1.37	
Maligt dysrhythmia	1.832	1.439	0.77	.441	.393	8.545	
Chronic obstructive pulmonary disease	1.046	.628	0.08	.94	.323	3.391	
Ischemic heart disease	.771	.277	-0.72	.47	.381	1.56	
Peripheral vascular disease	2.07	1.64	0.92	.359	.438	9.779	
Endocrine diseases	.895	.107	-0.92	.356	.708	1.132	
Gastrointestinal diseases	1.092	.121	0.79	.428	.879	1.356	
Stroke/transient ischemic attack	1.198	.39	0.55	.58	.633	2.267	
Anticoagulants	.832	.084	-1.81	.07	.682	1.015	*

Steroids	1	.	.	.	.	.	
Constant	1.549	.388	1.75	.081	.948	2.532	*

Mean dependent var	0.673	SD dependent var	0.469
Pseudo r-squared	0.033	Number of obs	1959.000
Chi-square	82.559	Prob > chi2	0.000
Akaike crit. (AIC)	2428.892	Bayesian crit. (BIC)	2529.335

*** p<.01, ** p<.05, * p<.1

Supplementary Table 4. Confounding balancing comparisons of PD-L1 users and PD-1 users after propensity score matching with 1:2 nearest neighbor search using Stata.

	**Mean**			**t-test**		
**Variable**	**Treated**	**Control**	**%bias**	**t**	**p>t**	**V(T)/V(C)**
Male gender	0.747	0.729	4	0.43	0.666	.
Baseline age, years	63.057	63.851	-6.5	-0.7	0.487	0.57*
Charlson standard comorbidity index	6.534	6.525	0.3	0.03	0.976	0.92
Hypertension	0.131	0.12	3.4	0.36	0.72	.

Liver diseases	0.036	0.041	-1.8	-0.25	0.805	.
Hip fractures/accident falls	0.059	0.041	8.5	0.87	0.383	.
Renal diseases	0.131	0.143	-3.2	-0.35	0.73	.
Diabetes mellitus	0.09	0.104	-4.9	-0.48	0.631	.
Maligt dysrhythmia	0	0	.	.	.	.
Chronic obstructive pulmonary disease	0.009	0.011	-2.5	-0.24	0.813	.
Ischemic heart disease	0.023	0.018	2.8	0.34	0.737	.
Peripheral vascular disease	0.005	0.005	0	0	1	.
Endocrine diseases	0.253	0.233	4.6	0.5	0.619	.
Gastrointestinal diseases	0.833	0.839	-1.6	-0.19	0.848	.
Stroke/transient ischemic attack	0.036	0.038	-1.2	-0.13	0.9	.
Anticoagulants	0.543	0.55	-1.4	-0.14	0.886	.
Steroids	0.543	0.55	-1.4	-0.14	0.886	.
* if variance ratio outside [0.77; 1.30]						
Ps R2	LR chi2	p>chi2	MeanBias			
0.005	3.03	1	3			
MedBias	B	R	%Var			
2.6	16.6	0.76	50			
* if B>25%, R outside [0.5; 2]						

Supplementary Table 5. Estimations of bootstrapped standard error (replications=50) that incorporates the propensity matching with 1:2 nearest neighbor search strategy.

Observed Coef.	Bootstrap Std. Err.	z	P>z	Normal based [95% Conf. Interval]
-0.1447964	0.0571656	-2.53	0.011	[-0.2568388, -0.032754]

Supplementary Table 6. Clinical characteristics of patients with/without mortality risk before and after 1:2 propensity score matching.

* for *SMD* 0.2; APTT: applied partial thromboplastin test; PD-1: Programmed death 1 inhibitors; PD-L1: programmed death 1 ligand inhibitors

Characteristics	Before matching		SMD	After 1:2 matching		SMD
	**All-cause mortality (N=1319)** Mean(SD);N or Count(%)	**Alive (N=640)** Mean(SD);N or Count(%)		**All-cause mortality (N=425)** Mean(SD);N or Count(%)	**Alive (N=238)** Mean(SD);N or Count(%)	
Demographics						
Male gender	892(67.62%)	449(70.15%)	0.05	318(74.82%)	180(75.63%)	0.02
Female gender	427(32.37%)	191(29.84%)	0.05	107(25.17%)	58(24.36%)	0.02
Baseline age, years	61.4(13.3);n=1319	60.2(14.5);n=640	0.09	63.2(10.2);n=425	62.7(10.2);n=238	0.05
<40	93(7.05%)	58(9.06%)	0.07	14(3.29%)	6(2.52%)	0.05
[40, 50)	134(10.15%)	60(9.37%)	0.03	29(6.82%)	14(5.88%)	0.04
[50-60)	326(24.71%)	155(24.21%)	0.01	93(21.88%)	62(26.05%)	0.1
[60-70)	421(31.91%)	210(32.81%)	0.02	186(43.76%)	102(42.85%)	0.02

[70-80)	265(20.09%)	126(19.68%)	0.01	87(20.47%)	46(19.32%)	0.03
>=80	80(6.06%)	31(4.84%)	0.05	16(3.76%)	8(3.36%)	0.02
Past comorbidities						
Charlson standard comorbidity index	6.4(3.3);n=1319	5.4(3.1);n=640	0.3*	7.0(3.1);n=425	5.6(2.9);n=238	0.45*
Hypertension	181(13.72%)	75(11.71%)	0.06	61(14.35%)	27(11.34%)	0.09
Liver diseases	144(10.91%)	49(7.65%)	0.11	18(4.23%)	6(2.52%)	0.09
Hip fractures/accident falls	57(4.32%)	20(3.12%)	0.06	19(4.47%)	18(7.56%)	0.13
Renal diseases	197(14.93%)	95(14.84%)	<0.01	54(12.70%)	31(13.02%)	0.01
Diabetes mellitus	107(8.11%)	49(7.65%)	0.02	38(8.94%)	22(9.24%)	0.01
Maligt dysrhythmia	10(0.75%)	2(0.31%)	0.06	0(0.00%)	2(0.84%)	0.13
Chronic obstructive pulmonary disease	11(0.83%)	4(0.62%)	0.02	5(1.17%)	1(0.42%)	0.09
Ischemic heart disease	40(3.03%)	21(3.28%)	0.01	8(1.88%)	7(2.94%)	0.07
Peripheral vascular disease	9(0.68%)	2(0.31%)	0.05	2(0.47%)	1(0.42%)	0.01
Endocrine diseases	377(28.58%)	171(26.71%)	0.04	111(26.11%)	55(23.10%)	0.07
Gastrointestinal diseases	960(72.78%)	452(70.62%)	0.05	362(85.17%)	189(79.41%)	0.15
Stroke/transient ischemic attack	56(4.24%)	24(3.75%)	0.03	17(4.00%)	7(2.94%)	0.06
Hospitalization						

Average readmission	70.8(200.0);n=1247	64.0(184.2);n=629	0.04	72.6(237.2);n=411	52.7(118.7);n=236	0.11
Total episode number	12.2(11.8);n=1247	18.3(16.6);n=629	0.43*	12.6(10.1);n=411	15.9(11.8);n=236	0.3*
Overall hospital stay, days	39.3(42.2);n=1247	27.1(26.9);n=629	0.34*	38.6(35.2);n=411	23.2(19.6);n=236	0.54*
Medications						
PD-L1 v.s. PD-1	115(8.71%)	106(16.56%)	0.24*	115(27.05%)	106(44.53%)	0.37*
PD-L1 expenditure, HKD	64470.8(52756.4);n=115	134899.3(121584.5);n=106	0.75*	64470.8(52756.4);n=115	134899.3(121584.5);n=106	0.75*
Total PD-L1 dose amount, mg	11141.0(23955.0);n=115	14274.9(30903.5);n=106	0.11	11141.0(23955.0);n=115	14274.9(30903.5);n=106	0.11
PD-L1 inhibitors duration, days	100.9(121.4);n=115	257.4(236.0);n=106	0.83*	100.9(121.4);n=115	257.4(236.0);n=106	0.83*
PD-1 expenditure	132406.3(227146.6);n=1204	329433.6(364585.4);n=546	0.65*	155528.6(204178.9);n=310	307662.2(326498.3);n=144	0.56*
Total PD-1 dose amount (MG)	2324.5(9779.2);n=1204	3905.8(11851.9);n=546	0.15	2114.9(5352.1);n=310	3186.2(4625.7);n=144	0.21*
PD-1 inhibitors duration, days	152.0(200.9);n=1204	314.5(272.0);n=546	0.68*	162.9(209.0);n=310	304.0(257.8);n=144	0.6*
Anticoagulants	727(55.11%)	381(59.53%)	0.09	233(54.82%)	129(54.20%)	0.01
Steroids	727(55.11%)	381(59.53%)	0.09	233(54.82%)	129(54.20%)	0.01
Inflamatory subclinical biomarkers						
Neutrophil-to-lymphocyte ratio	4.8(6.8);n=1316	4.2(5.5);n=636	0.09	4.6(7.5);n=425	3.7(4.0);n=238	0.15
Platelet-to-lymphocyte ratio	220.6(276.2);n=1317	195.2(156.2);n=636	0.11	226.7(365.3);n=425	180.8(136.5);n=238	0.17

Aspartate transaminase-to-alanine transaminase ratio	2.0(4.3);n=888	1.4(1.2);n=420	0.22*	1.6(2.5);n=254	1.2(0.7);n=151	0.24*
Triglyceride glucose index	7.05(0.61);n=394	7.07(0.66);n=186	0.03	7.0(0.6);n=134	7.1(0.7);n=73	0.07
Urea-to-creatinine ratio	73.4(39.8);n=1303	72.6(42.6);n=634	0.02	70.2(32.5);n=422	68.2(22.1);n=237	0.07
Monocyte-to-lymphocyte ratio	0.5(0.4);n=1314	0.4(0.6);n=636	0.06	0.5(0.4);n=424	0.4(0.4);n=238	0.19
Complete blood counts						
Mean corpuscular volume, fL	88.1(8.3);n=1317	87.8(8.0);n=636	0.04	88.3(7.6);n=425	88.1(8.1);n=238	0.02
Eosinophil, x10^9/L	0.17(0.26);n=1316	0.21(0.38);n=636	0.12	0.2(0.29);n=425	0.21(0.21);n=238	0.04
Lymphocyte, x10^9/L	1.5(0.9);n=1317	1.6(0.8);n=636	0.08	1.5(0.8);n=425	1.6(0.7);n=238	0.14
Metamyelocyte, x10^9/L	0.4(0.7);n=169	1.9(10.0);n=56	0.22*	0.3(0.4);n=57	0.5(0.7);n=15	0.36*
Monocyte, x10^9/L	0.54(0.3);n=1317	0.52(0.33);n=636	0.06	0.6(0.3);n=425	0.5(0.3);n=238	0.17
Neutrophil, x10^9/L	5.1(3.3);n=1317	4.9(3.3);n=636	0.07	5.1(3.1);n=425	4.8(3.3);n=238	0.1
White blood count, x10^9/L	7.5(6.2);n=1317	7.2(3.6);n=636	0.07	7.4(3.6);n=425	7.1(3.7);n=238	0.08
Mean cell haemoglobin, pg	30.7(3.4);n=1317	30.6(3.3);n=636	0.01	30.7(3.2);n=425	30.9(3.2);n=238	0.07
Myelocyte, x10^9/L	0.5(0.9);n=249	1.5(6.0);n=79	0.24*	0.4(0.8);n=76	1.5(2.6);n=21	0.58*
Platelet, x10^9/L	249.1(110.5);n=1317	245.6(104.4);n=636	0.03	259.0(103.0);n=425	241.3(90.2);n=238	0.18
Red blood count, x10^12/L	4.4(0.7);n=1317	4.5(0.7);n=636	0.14	4.4(0.7);n=425	4.6(0.6);n=238	0.22*
Hematocrit, L/L	0.38(0.06);n=1272	0.39(0.05);n=623	0.15	0.39(0.05);n=396	0.4(0.05);n=230	0.22*

Renal and liver functions						
K/Potassium, mmol/L	4.12(0.44);n=1306	4.14(0.43);n=635	0.06	4.12(0.43);n=423	4.15(0.42);n=238	0.06
Urate, mmol/L	0.33(0.15);n=419	0.32(0.12);n=208	0.04	0.34(0.14);n=111	0.33(0.1);n=75	0.05
Albumin, g/L	38.7(6.2);n=1305	40.2(5.1);n=634	0.26*	39.1(5.7);n=423	40.3(4.8);n=237	0.22*
Na/Sodium, mmol/L	138.8(4.1);n=1306	139.8(3.0);n=635	0.29*	138.9(3.7);n=423	139.8(2.7);n=238	0.28*
Urea, mmol/L	5.5(2.3);n=1303	5.6(2.6);n=634	0.02	5.2(1.8);n=422	5.5(1.7);n=237	0.14
Protein, g/L	71.5(13.6);n=1252	73.3(11.0);n=601	0.15	70.7(15.8);n=400	73.4(11.4);n=224	0.19
Bilirubin, umol/L	14.0(28.2);n=1306	10.8(17.5);n=635	0.14	11.1(13.2);n=423	11.7(26.5);n=238	0.03
Creatinine, umol/L	85.6(70.1);n=1317	83.5(42.1);n=635	0.04	85.2(76.7);n=424	84.2(30.8);n=238	0.02
SD of creatinine	41.9(116.3);n=1312	22.0(68.8);n=630	0.21*	33.6(78.2);n=422	22.8(88.9);n=236	0.13
Aspartate transaminase, U/L	60.5(119.5);n=936	41.4(61.1);n=435	0.2*	42.5(64.4);n=283	39.0(72.2);n=160	0.05
SD of aspartate transaminase	74.1(223.3);n=855	28.1(66.1);n=395	0.28*	41.0(91.7);n=263	28.3(68.4);n=146	0.16
Alkaline phosphatase, U/L	114.2(122.3);n=1306	97.3(84.6);n=635	0.16	103.2(104.3);n=423	100.0(91.8);n=238	0.03
SD of alkaline phosphatase	81.5(107.3);n=1298	30.4(47.3);n=630	0.62*	75.8(103.9);n=419	27.0(41.8);n=236	0.62*
Alanine transaminase, U/L	37.6(58.7);n=1257	34.6(49.1);n=619	0.06	30.7(27.5);n=394	32.7(32.8);n=229	0.07
SD of alanine transaminase	41.5(93.1);n=1244	26.0(63.1);n=611	0.2	30.7(50.8);n=388	23.9(53.0);n=224	0.13
Lipid, iron and calcium profile						
Total iron-binding capacity, L	40.8(12.8);n=86	41.9(9.9);n=17	0.1	40.6(13.6);n=17	47.4(7.8);n=5	0.62*

VitaminB12, pmol/L	425.7(314.0);n=39	560.6(372.1);n=10	0.39*	375.2(321.8);n=14	522.5(218.3);n=3	0.54*
Folate, ng/mL	21.4(10.1);n=56	23.2(10.9);n=13	0.18	22.5(11.8);n=13	15.8(3.9);n=6	0.76*
Ferritin, pmol/L	2833.9(5276.2);n=66	1598.8(2017.0);n=20	0.31*	2547.3(3542.8);n=14	880.3(1198.5);n=8	0.63*
Calcium, mmol/L	2.31(0.16);n=794	2.33(0.13);n=287	0.1	2.32(0.15);n=235	2.33(0.14);n=105	0.03
SD of calcium	0.1(0.07);n=740	0.08(0.04);n=247	0.32*	0.09(0.07);n=221	0.08(0.04);n=93	0.24*
Phosphate, mmol/L	1.05(0.23);n=703	1.07(0.19);n=245	0.09	1.1(0.2);n=203	1.0(0.2);n=83	0.11
SD of phosphate	0.2(0.1);n=595	0.1(0.1);n=190	0.37*	0.2(0.1);n=175	0.1(0.1);n=66	0.21*
Inorganic, mmol/L	6.8(8.8);n=202	5.0(7.9);n=57	0.21*	7.2(11.4);n=56	4.7(5.8);n=23	0.28*
SD of inorganic	2.13(3.39);n=86	2.11(3.9);n=27	0.01	1.0(1.6);n=29	0.4(0.6);n=12	0.43*
Glycemic and clotting profile						
Triglyceride, mmol/L	1.4(1.0);n=409	1.5(1.2);n=189	0.06	1.4(1.0);n=140	1.5(1.5);n=74	0.08
SD of triglyceride	0.35(0.53);n=202	0.36(0.53);n=92	0.02	0.4(0.5);n=52	0.43(0.7);n=38	0.06
Glucose, mmol/L	6.3(2.4);n=1214	6.2(1.9);n=559	0.05	6.2(2.3);n=397	6.4(2.0);n=211	0.06
SD of glucose	1.6(1.3);n=1135	1.3(1.2);n=503	0.18	1.6(1.5);n=372	1.3(1.1);n=192	0.2
HbA1c, g/dL	12.7(1.9);n=1317	13.0(1.9);n=636	0.12	12.9(1.8);n=425	13.3(1.9);n=238	0.22*
SD of HbA1c	1.4(0.5);n=1316	1.1(0.5);n=631	0.51*	1.4(0.5);n=425	1.2(0.5);n=235	0.47*
High sensitive troponin-I, ng/L	457.9(12369.6);n=897	15.7(71.2);n=244	0.05	41.6(359.0);n=285	15.7(38.6);n=87	0.1
SD of high sensitive	114.8(1195.8);n=612	163.5(1792.6);n=143	0.03	41.8(237.0);n=185	12.7(36.3);n=60	0.17

troponin-I						
APTT, second	30.9(5.9);n=528	30.6(3.9);n=166	0.06	30.5(4.0);n=160	30.3(3.5);n=57	0.04
SD of APTT	2.7(3.3);n=389	2.2(2.5);n=121	0.17	2.0(1.7);n=116	2.2(2.0);n=36	0.06
Lactate dehydrogenase, U/L	362.2(492.6);n=1014	231.6(95.5);n=417	0.37*	338.9(420.8);n=318	223.0(72.8);n=148	0.38*
SD of lactate dehydrogenase	195.0(461.6);n=819	53.5(48.1);n=321	0.43*	155.5(239.4);n=252	45.0(37.5);n=113	0.64*
Total cholesterol, mmol/L	4.57(1.07);n=410	4.6(1.01);n=189	0.02	4.7(1.2);n=140	4.5(0.9);n=74	0.25*
SD of total cholesterol	0.5(0.4);n=202	0.4(0.4);n=93	0.18	0.6(0.5);n=55	0.4(0.4);n=38	0.28*
Low-density lipoprotein, mmol/L	2.62(0.94);n=399	2.62(0.88);n=184	<0.01	2.8(1.1);n=137	2.5(0.7);n=71	0.32*
SD of low-density lipoprotein	0.43(0.37);n=192	0.38(0.37);n=91	0.13	0.44(0.44);n=51	0.37(0.32);n=37	0.17
High-density lipoprotein, mmol/L	1.3(0.4);n=405	1.4(0.4);n=188	0.06	1.31(0.37);n=139	1.32(0.39);n=73	0.01
SD of high-density lipoprotein	0.2(0.1);n=190	0.1(0.1);n=90	0.33*	0.2(0.2);n=49	0.1(0.1);n=38	0.19

Supplementary Table 7. Sensitivity analysis 1: Adjusted hazard ratios (and 95% CIs) of PD-L1 v.s. PD-1 with cause-specific and subdistribution hazard competing risk analysis models for new onset cardiac composite and mortality outcomes after 1:2 propensity score matching.

* for p≤ 0.05, ** for p ≤ 0.01, *** for p ≤ 0.001; HR: hazard ratio; CI: confidence interval.

**Model**	**Adverse outcomes**	**PD-L1 v.s. PD-1**<br>**HR [95% CI];p value**
Cause-specific hazard model	New onset cardiac composite	0.30[0.16, 0.57]; 0.0002***
	All-cause mortality	0.93[0.74- 1.17];0.525
Subdistribution hazard model	New onset cardiac composite	0.35[0.19, 0.55]; <0.0001***
	All-cause mortality	0.85[0.70- 0.93];0.0312*

Supplementary Table 8. Sensitivity analysis 2: Hazard ratios for associations of PD-L1 v.s. PD-1 using Cox proportional hazard model for adverse new onset cardiac composite and mortality outcome in the 1:2 matched cohort, with half-year lag time.

* for p≤ 0.05, ** for p ≤ 0.01, *** for p ≤ 0.001; HR: hazard ratio; CI: confidence interval.

**Adverse outcomes**	**PD-L1 v.s. PD-1**<br>**HR [95% CI];P value**
New onset cardiac composite	0.33[0.19, 0.59]; <0.0001***
All-cause mortality	0.83[0.75- 0.97];0.0225*

Supplementary Table 9. Sensitivity analysis 3: Risk of incident new onset cardiac composite and mortality outcomes associated with treatment of PD-L1 v.s. PD-1 with multiple matching adjustment approaches.

* for p≤ 0.05, ** for p ≤ 0.01, *** for p ≤ 0.001; HR: hazard ratio; CI: confidence interval;

PS: propensity score, HDPS: high dimensional propensity score, IPTW: inverse probability of treatment weighting.

**Outcome**	**HR after PS stratification [95% CI];P value**	**HR after HDPS matching [95% CI];P value**	**HR after PS IPTW [95% CI]; P value**
Composite outcome	0.34[0.17, 0.67]; 0.0003***	0.36[0.13, 0.65]; <0.0001***	0.34[0.16, 0.72]; 0.0001***
All-cause mortality	0.82[0.63- 0.95];0.0325*	0.81[0.54- 1.00];0.0045**	0.80[0.44- 0.96];0.0225*

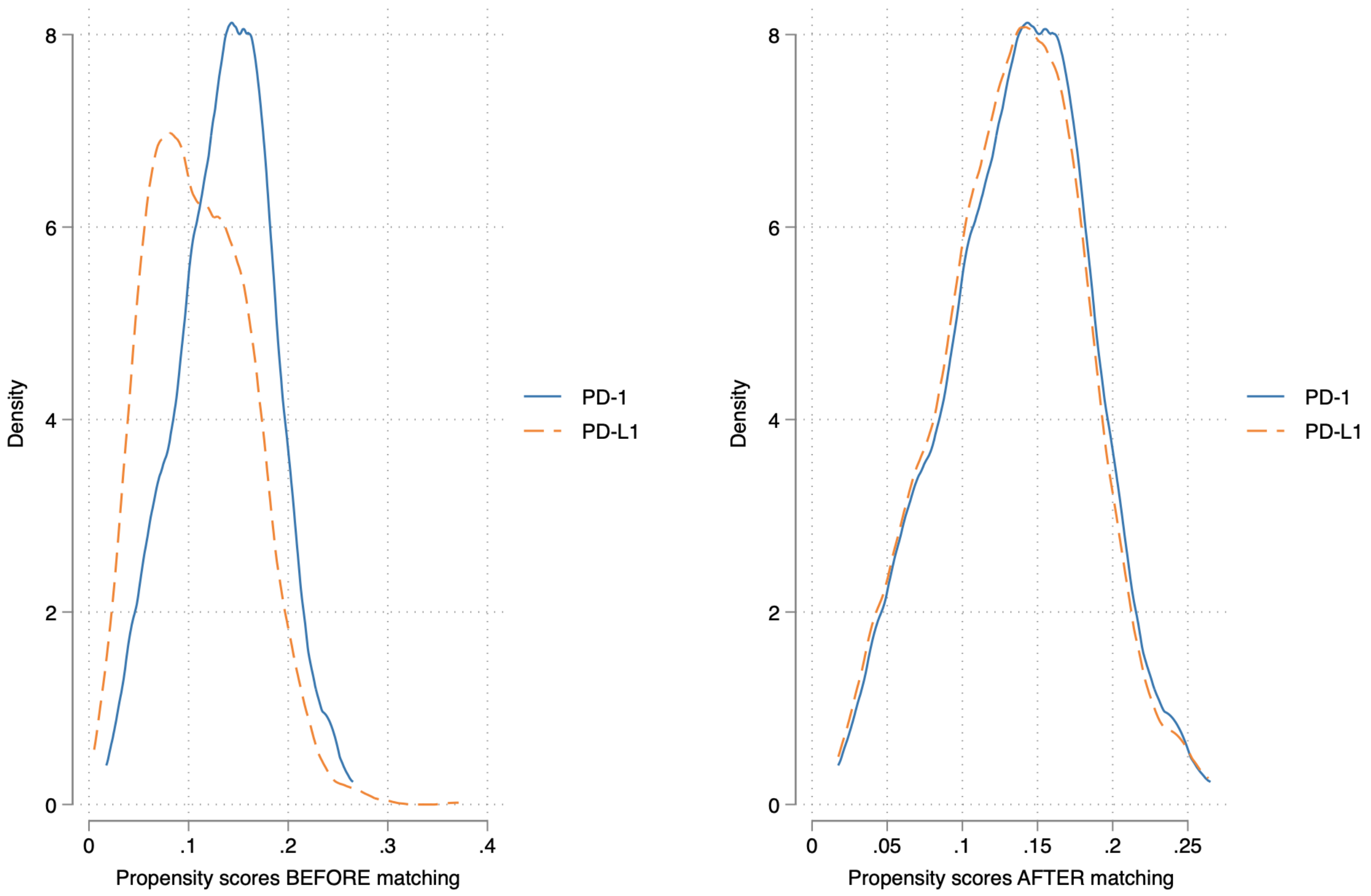

Supplementary Figure 1. Propensity score matching for PD-L1v.s. PD-1 before and after 1:2 matching with nearest neighbor search strategy.
